# Associations of different types of physical activity and sedentary behavior with self-rated health in children and adolescents: a systematic review of research from 2010 to 2024

**DOI:** 10.1186/s12966-025-01747-2

**Published:** 2025-04-21

**Authors:** Yahan Liang, Xihe Zhu, Ji-Hye Yang, Fei Wang, Danqing Zhang, Xingyi Yang, Sitong Chen, Yang Liu

**Affiliations:** 1 School of Physical Education, Shanghai University of Sport, Shanghai, 200438 China; 2 Department of Human Movement Studies and Special Education, Old Dominion University, Norfolk, VA 23529 USA; 3 Kunshan Lujia Senior High School, Kunshan, 215331 China; 4 Department of Sports and Health Sciences, Academy of Wellness and Human Development, Hong Kong Baptist University, Hong Kong, 999077 China; 5 Institute for Health and Sport, Victoria University, Melbourne, 3011 Australia; 6 Shanghai Research Center for Physical Fitness and Health of Children and Adolescents, Shanghai University of Sport, Shanghai, 200438 China

**Keywords:** Adolescence, Child, Exercise, Movement behavior, Screen time, Perceived health

## Abstract

**Background:**

Self-rated health (SRH) is one of the common measures to evaluate individuals’ overall health. Many studies have explored the associations between different types of physical activity (PA), sedentary behavior (SB), and SRH in children and adolescents. These studies report inconsistent findings and sometimes highlight gender differences. This systematic review aims to synthesize findings to provide a comprehensive evaluation of these associations.

**Methods:**

English-language articles published between January 2010 and September 2024 were systematically searched through Web of Science, PubMed, and EBSCOhost databases. Following PRISMA guidelines, we included 47 studies in this review that meet eligibility criteria. Study quality was assessed using the National Institutes of Health's study quality assessment tool for observational cohort and cross-sectional studies.

**Results:**

The majority of study findings show that ≥ 60 min/day of moderate-to-vigorous PA (≥ 4 days/week), vigorous PA (≥ 3 days/week or ≥ 4 times/week), out-of-school PA (≥ 2 days/week), and sports participation are significantly positive associated with SRH. Additionally, evidence suggests that SB (e.g., watching TV and doing homework) generally shows no significant association with SRH. More study findings support that PA is positively associated with boys' SRH than that of girls.

**Conclusions:**

Findings show significant positive associations between PA and SRH, particularly those of vigorous and moderate-to-vigorous intensity, while the association between SB and SRH requires further investigation beyond TV and video game/computer times. Evidence of gender differences in the associations suggests the potential need for targeted strategies to enhance SRH in girls.

**Supplementary Information:**

The online version contains supplementary material available at 10.1186/s12966-025-01747-2.

## Background

Self-rated health (SRH) reflects an individual's overall health perception, typically measured with a single question asking respondents to rate their overall health [1]. SRH is widely used and is an important concept in research, prevention, and clinical medicine [1], and it consistently aligns with objective health measures [2]. For example, SRH is frequently measured in global surveys across various age groups [1, 3, 4], including children and adolescents, such as the Health Behavior in School-aged Children (HBSC) study conducted in 45 countries [3]. Among children and adolescents, SRH is related to various medical, psychological, social, and lifestyle factors [5–10]. Research has shown that healthy behaviors such as non-smoking, non-drinking, and healthy eating habits are associated with good SRH in children and adolescents [11–13]. Conversely, obesity, loneliness, hopelessness, and a lack of happiness are associated with poor SRH among this demographic [6, 14, 15]. Research has shown that SRH is a reliable predictor of both current and future physical and mental health among adolescents, as well as future morbidity [6, 15, 16]. Therefore, enhancing SRH in childhood and adolescence is associated with better current health status, which in turn may contribute to improved long-term health outcomes and reduced morbidity in adulthood.

From 1990 to 2017, children and adolescent deaths from communicable diseases decreased, while noncommunicable diseases emerged as a major global health burden [17]. Lack of physical activity (PA) is one of the top four risk factors for global mortality related to noncommunicable diseases [18], and sedentary behavior (SB), such as screen time, is a critical factor affecting children's and adolescents' physical and mental health [19]. The World Health Organization and Canadian Society for Exercise Physiology recommend that children and adolescents aged 5–17 engage in at least 60 min of moderate to vigorous PA (MVPA) [19, 20] and no more than 2 h of recreational screen time daily [20]. Given the close connection between PA, SB, and health, numerous studies have examined the association between PA, SB and SRH in children and adolescents [21–24].

Previous studies often suggest that those engaged in MVPA are more likely to report good SRH [22–24]. However, some studies report conflicting findings with no significant association between MVPA and SRH [11, 25]. Additionally, studies have investigated the association between PA and SRH in different PA contexts (e.g., school-based and outside of school) [22, 26]. For example, Curtin et al. (2018) found no significant association between school-based or out-of-school PA and adolescents' SRH [26], while others reported that out-of-school vigorous PA (VPA) was significantly associated with better SRH in girls [22]. Studies have also explored associations between different modalities of PA and SRH [8, 12]. Husu et al. (2016) found that children and adolescents who took more daily steps were more likely to rate their health as excellent compared to those with fewer steps [8], and Hwang & Kye (2018) reported that adolescents who engaged in muscle-strengthening exercises three or more days per week had better SRH than those who did not [12].

Studies that investigated the association between SB and SRH in children and adolescents often focused on screen time [14, 23, 27]. For example, Moor et al. (2014) found that those who spent more time watching TV or playing computer games were more likely to report poorer SRH [23]. In contrast, Meireles et al. (2015) found no significant association between those types of screen time and SRH [27]. Additionally, research has explored the association between study time (e.g. homework) and SRH [14, 28]. Herman et al. (2014) found that longer reading time was associated with better SRH in girls, but no significant association was found in boys [14]. Martinez-Lopez et al. (2015) found that homework time was not associated with adolescents’ SRH [28]. In summary, studies have explored the associations of different types of PA and SB with SRH in children and adolescents, and they reported inconsistent findings and gender differences in these associations. These findings underscore the need for research to systematically identify, evaluate, and synthesize findings from these studies to provide a comprehensive literature summary.

While a systematic review of studies on the association between PA, SB, and SRH in children and adolescents was conducted five years ago [21], it has several limitations. The review broadly examines overall associations between PA and SRH but overlooks detailed analyses of specific PA types, which are essential for identifying targeted interventions. Similarly, it examines only a limited range of SB types—TV, computers/video games, and total screen time—while ignoring significant SB, such as homework that greatly contributes to children and adolescents’ sedentary time. This limited scope risks missing important distinctions in how different types of PA and SB are associated with SRH. Additionally, the review included only 45% of PA studies and 37% of SB studies in their meta-analysis, potentially excluding relevant insights. An updated review is essential to ensure that conclusions and recommendations are relevant to current practices and knowledge. This review aims to provide a more detailed synthesis, exploring the associations between different types of PA and SB with SRH among children and adolescents, offering clearer direction and specific practical strategies to promote health in children and adolescents.

## Methods

### Data sources and search strategy

This systematic review was conducted in accordance with the Preferred Reporting Items for Systematic Reviews and Meta-Analyses (PRISMA) guidelines [29]. A comprehensive literature search was performed across the Web of Science, PubMed, and EBSCOhost databases, covering publications from January 2010 to September 2024. Additionally, citation searches from a previous review were performed [21], and eligible studies identified through this process were included. As seen in Table 1, the search strategy focused on three main topics: (1) Exposure: PA or SB; (2) Outcome: SRH; and (3) Participants: children or adolescents. The final search queries combined these topics as (1) AND (2) AND (3). The search strategy was collaboratively established by the first author and the corresponding author. Detailed keywords and search strategies are provided in Table 1. All retrieved literature was organized and screened using EndNote 20.6. The first author initially identified and screened the studies for inclusion, then distributed the retrieved literature to the third (60%) and sixth authors (40%) to screen independently to minimize potentially overlooked studies, achieving an agreement rate of 85.1%. Discrepancies during the screening process were resolved through discussion between authors to ensure consistent and unbiased selection according to the eligibility criteria below.

**Table 1:** Keywords and search strategy

Search query	Search topic	Search keywords
1	Outcome: self-rated health	"self-assessed health" OR "self-assessments of health" OR "self-rated health" OR "self-ratings of health" OR "self-perceived health" OR "perceived health" OR "self-perceptions of health" OR "self-evaluated health" OR "self-evaluations of health" OR "self-reported health" OR "self-report health" OR "health indicator"
2	Exposure: a. physical activity. b. sedentary behavior	activity OR activities OR "physical activity" OR "physical activities" OR "motor activity" OR "motor activities" OR "outdoor activity" OR "outdoor activities" OR "locomotor activity" OR "locomotor activities" OR "activity behavior" OR "organized activities" OR "organized activity" OR behavior OR locomotion OR exercise OR "physical exercise" OR "exercise behavior" OR sport OR sedentary OR "sedentary behavior" OR "sedentary lifestyle" OR sedentariness OR "physical inactivity" OR "physically inactive" OR sitting OR recumbency OR reclining OR lying OR media OR medium OR computer OR PC OR television OR TV OR laptop OR tablet OR iPad OR phone OR video OR "internet use" OR "social media" OR game OR "electronic game" OR "e-game" OR "video game" OR gaming OR "screen time" OR screen OR "screen-based media use" OR "screen-based activities" OR "screen-based activity" OR "screen-based behavior" OR homework OR reading OR lifestyle OR "life style" OR "behavioral factor" OR "socio-environmental" OR "socio-demographic"
3	Participants: a. children. b. adolescents	adolescent OR adolescence OR young OR youngster OR children OR childhood OR child OR school OR schooler OR teenage OR teenager OR teen OR juvenile OR girl OR boy OR kid OR youth OR student OR pupil
Final search query	Intersection of three topics	1 AND 2 AND 3

In our search strategy, we used different fields for each database. In Web of Science, we searched the outcome variable under 'Topic' and the exposure and participant variables under 'Title'. Since EBSCOhost and PubMed do not have a 'Topic' field, we searched for the outcome variable in 'Subject Terms' in EBSCOhost and in 'Title/Abstract' in PubMed

### Eligibility criteria

Inclusion criteria for article screening were: (1) studies involving children and adolescents aged 5–17, as defined by the World Health Organization guidelines [19]. Baseline sample age was used for longitudinal studies. If age was not reported, school grade (primary, middle, or high school) was used as a proxy; (2) peer-reviewed journal articles published between January 2010 and September 2024; (3) articles published in English; (4) reporting the results of associations of PA and/or SB with SRH; (5) studies with a clear distinction between PA and SB as independent variables and SRH as the dependent variable.

Exclusion criteria were: (1) published protocols, conference papers, reviews, commentaries, dissertations, letters, abstracts, and qualitative studies; (2) studies involving children and adolescents with physical disabilities or mental disorders; (3) duplicated studies across databases.

### Data extraction and data items

Data extraction was conducted using Microsoft Excel 16.89.1, where a standardized data table was developed to systematically extract and summarize information from the included studies. The extracted data included the following items: (1) basic information about the studies: listed authors, year of publication, and research design; (2) sample characteristics: sample size in analysis, the proportion of females, and age or grade range; if unavailable, the mean age was used; (3) measurement methods: PA and SB measurement tool (questionnaires and device-based measures); (4) results: findings on association of PA and/or SB with SRH in children and adolescents.

To ensure consistency, specific rules were applied when extracting data: in studies with multiple statistical methods (e.g., chi-square and logistic regression), regression results were prioritized. When multiple models were provided, we selected the model with the most control variables. If studies reported associations for multiple SRH categories (e.g., good vs. moderate/poor SRH and very good vs. moderate/poor SRH), we prioritized the latter [30]. As gender differences were a focus, results by gender were prioritized over other categories, such as ethnicity [31]. To avoid duplication, we did not extract aggregated variables for a summary of findings. For example, Liang et al. (2024) reported screen time on weekdays, weekends, and the whole week, since the whole week was the aggregate of weekdays and weekends, we included only weekday and weekend results [32]. Data extraction was performed by the first author, with the third (36%) and sixth authors (64%) independently repeating the process to ensure accuracy and consistency. The extracted findings were cross-checked by the first and third authors, with an overall agreement rate of 90.0%. Any disagreements were resolved through discussion, ensuring a robust and reliable extraction process.

### Evaluation of study quality

We evaluated study quality using the National Institutes of Health's study quality assessment tool, specifically designed for observational cohort and cross-sectional studies [33]. It is designed to evaluate the internal validity of studies by focusing on key concepts such as research sample size, the reliability and validity of variable measurement, and statistical analyses [33]. The tool comprises 14 evaluation items, each item scoring 1 point if the study meets the stated criteria and 0 points if it does not. The total score, ranging from 0 to 14, categorizes studies as low quality (0–4 points), medium quality (5–9 points), or high quality (10–14 points). At least two authors (first, third, fourth, and fifth) independently assessed and scored each study. Discrepancies in scoring were resolved through discussion to ensure consistency and accuracy in the final quality assessment.

### Coding of studies and summary

We summarized PA and SRH findings by the categories of PA intensity level (e.g., light and vigorous), context (in-school and out-of-school), and modality (e.g., muscle-strengthening exercises and steps). PA measures that did not clearly fit any one of these categories or were ambiguous were grouped under “Others.” SB and SRH findings were grouped through SB measures of recreational screen time and study time, with unclear or mixed SB types being grouped under the "Others" category. The frequency of reported associations of PA, SB, and SRH were aggregated based on the extracted findings from the included studies (see Supplementary Tables 1 and 2). Additionally, we also summarized these associations in longitudinal studies (evaluated as higher quality) and across different genders. To maintain consistency across studies with varying definitions of variables, we followed the World Health Organization’s definitions of PA and SB categories (i.e., PA intensity and recreational screen time) [19] and checked the PA context and modality by reviewing measurement details. For example, Jodkowska et al. (2019) reported VPA, but since the actual question referred to out-of-school VPA, we used the latter in our analysis [22]. Measurement details for each study are shown in Supplementary Table 3.

Following the method used in previous systematic reviews [34–36], we used the semi-quantitative review method to summarize the findings [36]. The association between each type of PA and SB with SRH in children and adolescents was determined by the percentage of supporting findings:(a) no association (code as “O”): 0–33% of findings reported a significant association (positive or negative association); (b) inconclusive association (code as “?”): 34–59% of findings reported a significant association; (c) positive (code as “ + ”) or negative (code as “–”) association: 60–100% of findings reported a significant positive or negative association, respectively [36]. These thresholds were chosen to align with previous systematic reviews utilizing semi-quantitative coding methods [34–36]. Additionally, when four or more findings supported the same direction of the association, a double-signed code was applied: no association (code as “OO”), inconclusive association (code as “??”), positive association (code as “ + + ”), and negative association (code as “– –”) [36]. To be consistent, we used *p* < 0.05 as the significance level across all included studies.

## Results

### Included study characteristics and quality evaluation

**Fig. 1:**
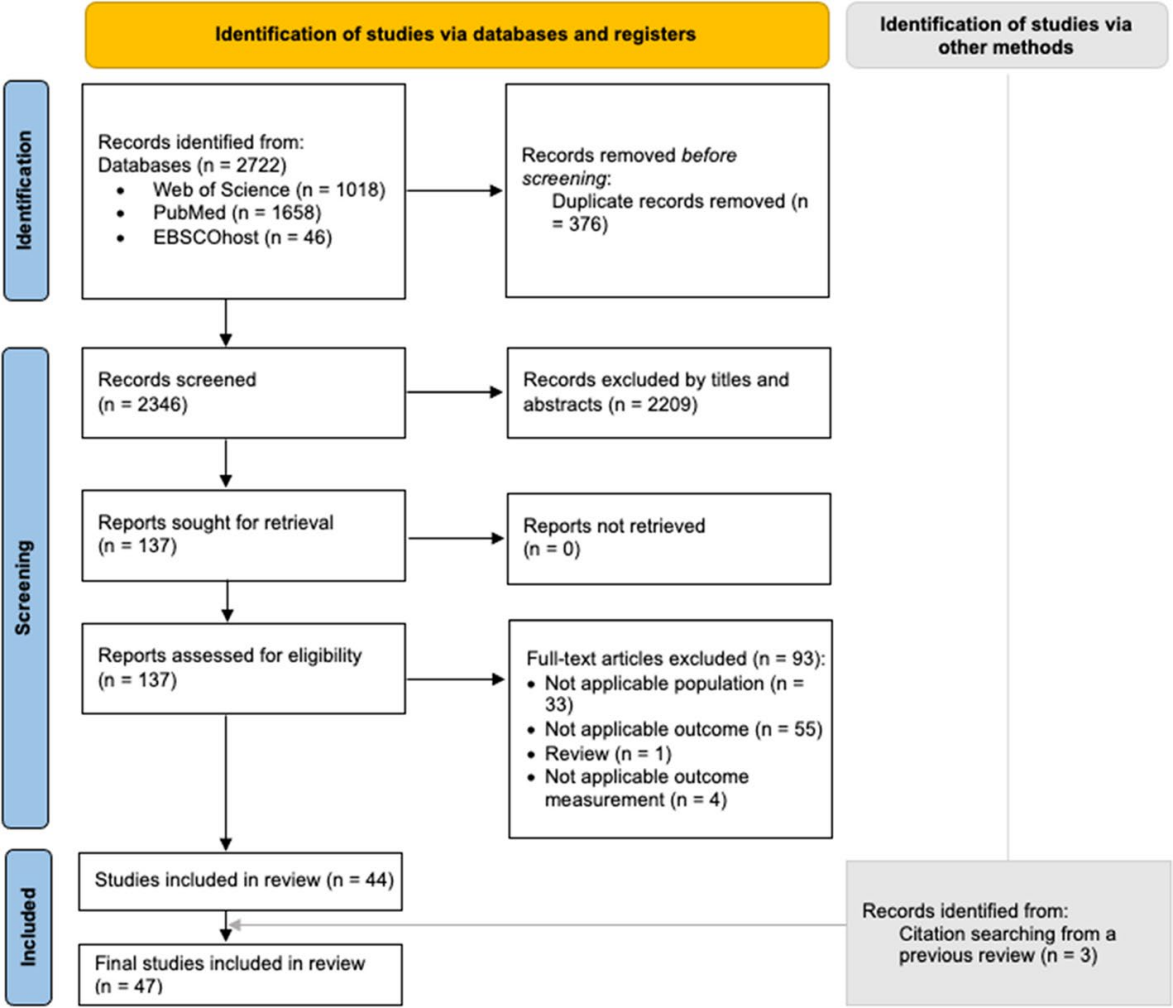
PRISMA flowchart for study selection

Following the identification and screening process (Fig. 1), we identified 2,722 studies in total, and 47 of them met the inclusion criteria and were included. Most included studies (*n* = 41, 87%) utilized a cross-sectional design, while six (13%) adopted a longitudinal design. Participants' ages ranged from 6 to 17 years, with sample sizes varying widely from 245 to 166,590 individuals. Most studies (*n* = 44, 94%) assessed PA and SB through questionnaires, while three used accelerometers to measure PA and two used accelerometers for SB assessment (Table 2).

**Table 2:** Characteristics of the included studies (*n* = 47)

Author, publish year	Sample size (F%) Age or grade	Measuring tool		Findings	
		PA	SB	PA and SRH (frequency or duration, if available)	SB and SRH (frequency or duration, if available)
**Cross-section studies**					
Park, 2024 [37]	166,590 (48.3%) 7–12 grades	Q	Q	PA: in 2019 ( +), 2020 ( +), 2021 ( +)	SB for study purpose: in 2019 (-), 2020 (-), 2021(-) SB for purpose other than study: in 2019 (-), 2020 (-), 2021(-)
Moran et al., 2024 [38]	11,859 (49.9%) 13–15 years	Q	NA	MVPA (≥ 1 h/d): 1–2 d/wk ( +), 3–4 d/wk ( +), 5–6 d/wk ( +), 7 d/wk ( +)	NA
Liang et al., 2024 [32]	4227 (52.12%) 8–17 years	Q	Q	M: MVPA (≥ 1 h/d): weekdays ( +), weekends ( +); school-based PA (> 1 h/d) ( +); extracurricular VPA (> 1 h/d) ( +) F: MVPA (≥ 1 h/d): weekdays ( +), weekends ( +); school-based PA (> 1 h/d) (O); extracurricular VPA (> 1 h/d) ( +)	M: Recreational screen time (> 2 h/d): weekdays (O)^a^, weekends (O)^a^; homework (> 2h/d): weekdays (O)^a^, weekends (O)^a^ F: Recreational screen time (> 2h/d): weekdays (-)^a^, weekends (-)^a^; homework (> 2h/d): weekdays (O)^a^, weekends (O)^a^
Du et al., 2024 [39]	7993 (47.5%) 10–15 years	NA	Q	NA	Internet use (-)^b^
de Sales et al., 2024 [40]	1182 (48.5%) 11–17 years	Q	NA	MVPA ( +)	NA
Gonzalez-Alvarez et al., 2023 [41]	1250 (49.9%) 12–17 years	Q	Q	PA: 1–2 times/wk (O), 3–4 times/wk (O), 5–6 times/wk ( +), ≥ 7 times/wk ( +)	SB: little time (O), moderate time (O), lot of time (O)
Wang et al., 2023 [42]	426 7–12 grades	Q	NA	MVPA ( +)	NA
Shi et al., 2023 [43]	2407 (46.8%) 10–17 years	Q	Q	MVPA (≥ 1 h/d) ( +)	Recreational screen time (> 2h/d) (O)^c^
Yang et al., 2023 [44]	8213 (48.1%) 8 grade	Q	Q	Exercise: 0.25–0.5 h/d ( +), > 0.5 h/d ( +); housework: 0.5–1 h/d (O), > 1 h/d ( +)	TV: 1–2 h/d ( +), > 2 h/d ( +); online and playing games: 1–2 h/d (-), > 2 h/d (-); homework time on school: 2–4 h/d (O), > 4 h/d (O); homework time off school: 2–4 h/d (O), > 4 h/d (-)
Kyan et al., 2022 [10]	6768 (50.7%) 10–14 years	Q	Q	MVPA (≥ 1 h/d): elementary school students (O), junior high school students ( +)	Recreational screen time (> 2h/d): elementary school students (O)^c^, junior high school students (O)^c^
Karchynskaya et al., 2022 [45]	888 (44.0%) 11–15 years	Q	NA	MVPA (≥ 1 h/d): 7 d/wk ( +), 5–7 d/wk ( +); organized leisure time sport activity ( +)	NA
Wang et al., 2022 [46]	116,828 (51.8%) 11–15 years	Q	NA	VPA: 7 times/wk ( +)^d^	NA
de Fátima Guimarães et al., 2022 [47]	263 (100%) 12–17 years	Q	Q	F: MVPA (≥ 1 h/d) ( +)	F: Recreational screen time (> 2h/d) (-)
Pierannunzio et al., 2022 [48]	58,976 (49.4%) 11,13 and15 years	Q	NA	M: MVPA (≥ 1 h/d, ≥ 4 d/wk): 11 ( +), 13 ( +), 15 ( +) years old; out-of-school VPA (≥ 2 d/wk): 11 (O), 13 ( +), 15 ( +) years old F: MVPA (≥ 1 h/d, ≥ 4 d/wk): 11 (O), 13 ( +), 15 ( +) years old; out-of-school VPA (≥ 2 d/wk): 11 ( +), 13 ( +), 15 ( +) years old	NA
Badura et al., 2021 [49]	45,900 11,13 and15 years	Q	NA	Organized leisure time sports activity ( +)	NA
Tebar et al., 2021 [50]	100,873 (51.9%) 14.3 years	Q	Q	M: PA (≥ 300 min/wk) ( +) F: PA (≥ 300 min/wk) (O)	M: SB (≥ 4 h/d) (-)^e^ F: SB (≥ 4 h/d) (-)^e^
Moral-García et al., 2020 [51]	516 (48.1%) 12–16 years	Q	NA	PA ( +)	NA
Marques et al., 2019 [52]	5024 (52.8%) 10–17 years	Q	Q	PA (≥ 1 h/d) ( +)	Recreational screen time (≥ 2 h/d) (O)
Jodkowska et al., 2019 [22]	1173 (100%) 15 years	Q	Q	F: Physical education (attend all and almost all classes) ( +), out-of-school VPA (≥ 2 d/wk) ( +), MVPA (≥ 1 h/d) ( +)	F: Recreational screen time (> 2 h/d) (-)^f^, social media (> 2 h/d) (-)^f^
Werneck et al., 2018 [53]	984 (58.8%) 10–17 years	NA	Q	NA	M: Computer and video game (-), TV (O) F: Computer and video game (O), TV (O)
Hwang et al., 2018 [12]	104,750 (46.1%) 7–12 grades	Q	NA	VPA (≥ 3 d/wk) ( +)^g^, muscle strengthening activity (≥ 3 days/wk) ( +)^g^, moderate PA (≥ 5 d/wk) (O)^g^	NA
Li et al., 2018 [9]	4966 (50.7%) 15–16 years	Q	Q	M: PA (≥ 3 times/wk) (O)^h1^ F: PA (≥ 3 times/wk) (O)^h1^	M: TV: 2–3 h/d (O)^h2^, 3–4 h/d (O)^h2^, > 4 h/d (O)^h2^; video games: 0–1 h/d (O)^h2^, 1–2 h/d (O)^h2^, 2–3 h/d (O)^h2^, > 3 h/d (O)^h2^; telephone: 0–0.5 h/d (O)^h2^, 0.5–1 h/d (O)^h2^, > 1 h/d (O)^h2^; mobile phone email use: 2–3 h/d (O)^h2^, 3–4 h/d (O)^h2^, > 4 h/d (O)^h2^; computer: 2–3 h/d (O)^h2^, 3–4 h/d (O)^h2^, > 4 h/d (O)^h2^ F: TV: 2–3 h/d (O)^h2^, 3–4 h/d ( +)^h2^, > 4 h/d (O)^h2^; video games: 0–1 h/d (-)^h2^, 1–2 h/d (O)^h2^, 2–3 h/d (O)^h2^, > 3 h/d (O)^h2^; telephone: 0–0.5 h/d (O)^h2^, 0.5–1 h/d (O)^h2^, > 1 h/d (O)^h2^; mobile phone email use: 2–3 h/d (O)^h2^, 3–4 h/d (O)^h2^, > 4 h/d (-)^h2^; computer: 2–3 h/d (O)^h2^, 3–4 h/d (O)^h2^, > 4 h/d (-)^h2^
Curtin et al., 2018 [26]	832 (49.8%) 10–17 years	Q	NA	School-based organized sports (≥ 1 times/wk) (O), after school/saturday school-based organized sport (≥ 1 times/wk) (O), sport outside of school (≥ 1 times/wk) (O)	NA
Silva et al., 2017 [54]	1342 10–17 years	NA	Q	NA	M: TV in weekdays: 2.1–4.0 h/d (O)^i^, > 4.0 h/d (O)^i^; TV in weekend: 2.1–4.0 h/d (-)^i^, > 4.0 h/d (-)^i^; video games/computer in weekdays: 0.1–2.0 h/d (O)^i^, 2.1–4.0 h/d (O)^i^, > 4.0 h/d (O)^i^; video games/computer in weekend: 0.1–2.0 h/d (O)^i^, 2.1–4.0 h/d (O)^i^, > 4.0 h/d (O)^i^ F: TV in weekdays: 2.1–4.0 h/d (O)^i^, > 4.0 h/d (O)^i^; TV in weekend: 2.1–4.0 h/d (O)^i^, > 4.0 h/d (O)^i^; video games/computer in weekdays: 0.1–2.0 h/d ( +)^i^, 2.1–4.0 h/d ( +)^i^, > 4.0 h/d (O)^i^; video games/computer in weekend: 0.1–2.0 h/d ( +)^i^, 2.1–4.0 h/d (O)^i^, > 4.0 h/d (O)^i^
Lachytova et al., 2017 [55]	1111 (47.2%) 14–16 years	Q	Q	VPA: 1 times/wk (O), 2–3 times/wk (O), 4–6 times/wk ( +), everyday ( +)	TV (≥ 2 h/d) (-)^j^, computer (≥ 2 h/d) (O)^j^
Granger et al., 2017 [56]	12,770 (51.4%) 15 years	Q	Q	MVPA (≥ 1 h/d) ( +)	Recreational screen time (≥ 4 h/d) (O)^k^
Husu et al., 2016 [8]	851 (52.0%) 7–14 years	A	A	Steps ( +), light PA ( +), MVPA (O)	SB (-)
Smith et al., 2015 [57]	1689 (46.8%) 11–12 years	Q	Q	Out-of-school PA (O)^l1^	SB (O)^l2^
Meireles et al., 2015 [27]	974 11–17 years	Q	Q	Active PA (≥ 300 min/wk) (O)^m^, active PA (≥ 300 min/wk) ( +)^m^	TV (O), video games/computer (O)
Martinez-Lopez et al., 2015 [28]	2293 (50.2%) 12–16 years	Q	Q	M: MVPA (≥ 1 h/d, > 4 d/wk) ( +)^n1^ F: MVPA (≥ 1 h/d, > 4 d/wk) ( +)^n1^	M: TV (≥ 4 h/d): weekdays (O)^n2^, weekend (O)^n2^; computer (≥ 4 h/d): weekdays (-)^n2^, weekend (O)^n2^; homework (≥ 4 h/d): weekdays (O)^n2^, weekend (O)^n2^ F: TV (≥ 4 h/d): weekdays (O)^n2^, weekend (O)^n2^; computer (≥ 4 h/d): weekdays (O)^n2^, weekend (O)^n2^; homework (≥ 4 h/d): weekdays (O) ^n2^, weekend (O)^n2^
Kantomaa et al., 2015 [30]	2229 (48.4%) 15–16 years	Q	NA	M: Out-of-school PA: the middle tertile (O), the highest tertile ( +) metabolic equivalent of task h/wk F: Out-of-school PA: the middle tertile (O), the highest tertile ( +) metabolic equivalent of task h/wk	NA
Herman et al., 2015 [58]	7725 (49%) 12–17 years	Q	Q	M: PA: ≥ 3.0 kilocalories per kilogram per day ( +)^o1^ F: PA: ≥ 3.0 kilocalories per kilogram per day ( +)^o1^	M: Recreational screen time (> 2 h/d) (-)^o2^ F: Recreational screen time (> 2 h/d) (-)^o2^
Badura et al., 2015 [59]	10,503 (50.8%) 11,13 and15 years	Q	NA	Individual sports ( +), team sports ( +)	NA
Moor et al., 2014 [23]	117,460 (53.3%) 11–15 years	Q	Q	MVPA (≥ 1 h/d, ≥ 5 d/wk) ( +)^p1^	TV (> 2 h/d) (-)^p2^, computer games (> 2 h/d) (-)^p2^, computer (> 2 h/d) (-)^p2^
Herman et al., 2014 [14]	527 (46.3%) 8–10 years	A	Q & A	M: VPA ( +), MVPA (≥ 1 h/d) ( +)^q1^, light PA (O) F: VPA (O), MVPA (≥ 1 h/d) (O)^q1^, light PA (O)	M: Video games/computer (> 2 h/d) (O)^q2^, reading (> 1 h/d) (O)^q2^, TV (> 2 h/d) (O)^q2^, homework (> 1 h/d) (O)^q2^, SB (O)^q2^ F: Video games/computer (> 2 h/d) (-)^q2^, reading (> 1 h/d) ( +)^q2^, TV (> 2 h/d) (O)^q2^, homework (> 1 h/d) (O)^q2^, SB (O)^q2^
Spein et al., 2013 [60]	598 15–16 years	Q	NA	Out-of-school VPA (≥ 1 times/wk) in Sami ( +)^r^, VPA (everyday) in Inuit ( +)^r^	NA
Afridi et al., 2013 [25]	414 (46.1%) 14–17 years	Q	NA	MVPA (≥ 3 d/wk) (O)^s^	NA
Tabak et al., 2012 [24]	600 (50.8%) 13 years	Q	Q	MVPA: Rural ( +)^t1^, urban ( +)^t1^	TV: rural (O)^t2^, urban (O)^t2^; computer: rural (O)^t2^, urban (O)^t2^
Richter et al., 2012 [61]	6997 (49.9%) 11–15 years	Q	NA	M: MVPA (≥ 1 h/d, ≥ 6 d/wk) (O)^u^ F: MVPA (≥ 1 h/d, ≥ 6 d/wk) ( +)^u^	NA
Zullig et al., 2011 [62]	245 (55.5%) 7–8 grades	Q	Q	M: Sports team ( +)^v1^, VPA (≥ 1d/wk) (O)^v1^, physical education (≥ 1d/wk) (O)^v1^ F: Sports team ( +)^v1^, VPA (≥ 1d/wk) (O)^v1^, physical education (≥ 1d/wk) (O)^v1^	M: TV (≥ 1 h/d) (O)^v2^ F: TV (≥ 1 h/d) (O)^v2^
Foti et al., 2010) [31]	12,193 9–12 grades	Q	NA	M: MVPA (≥ 1 h/d) ( +)^w1^ F: MVPA (≥ 1 h/d) (O)^w1^	TV (≥ 3 h/d): Non-Hispanic white (-)^w2^, Non-Hispanic black (O)^w2^, Hispanic (O)^w2^; computers (≥ 3 h/d): Non-Hispanic white (-)^w2^, Non-Hispanic black (O)^w2^, Hispanic (-)^w2^
**Longititunal studies**					
Joensuu et al., 2024 [63]	249 (41.8%) 7 grade	Q & A	NA	M: Self-reported PA ( +), accelerometer based MVPA (O) F: Self-reported PA (O), accelerometer based MVPA (O)	NA
Nigg et al., 2015 [11]	334 (55.1%) 9–12 years	Q	Q	MVPA (O)	Recreational screen time (O)
Liu et al., 2015 [64]	5238 (51.8%) 6–12 years	Q	NA	M: Outdoor PA ( +) F: Outdoor PA (O)	NA
Spengler et al., 2014 [65]	953 (54.5%) 11–17 years	Q	NA	M: PA ( +) F: PA (O)	M: Recreational screen time (O) F: Recreational screen time (O)
Jerdén et al., 2011 [66]	788 12–13 years	Q	NA	M: Out-of-school VPA (> 3times/wk) ( +), out-of-school PA (> 3times/wk) (O) F: Out-of-school VPA (> 3times/wk) ( +), out-of-school PA (> 3times/wk) (O)	NA
Elinder et al., 2011 [67]	2489 (51.8%) 15 years	Q	NA	M: VPA: > 4 h/wk ( +)^x^ F: VPA: > 4 h/wk (O)^x^	NA

*PA* physical activity, *SB* sedentary behavior, *VPA* vigorous physical activity, *MVPA* moderate to vigorous physical activity, *M* male, *F* female, *Q* questionnaire, *A* accelerometer, *NA* not applicable, *d* day, *wk* week, *h* hour

The findings in Table 2 showed the associations between higher levels of PA/SB and better SRH: significant positive association (“ + ”), significant negative association (“-”), or no significant association (“O”). To ensure consistency in the analysis, we standardized the presentation of results from those that reported data differently (e.g. higher SB positively associated with poorer SRH). Their original findings are as follows (see Supplementary Table 3 for details):

^a^ Compared to > 2 h/d of recreational screen time (reference group), ≤ 2 h/d of recreational screen time was significantly positively associated with better SRH in girls, not boys. Compared to > 2 h/d of homework, ≤ 2 h/d of homework had no significant association with better SRH in boys and girls

^b^ internet use was significantly positively associated with poorer SRH

^c^ Compared to > 2 h/d of recreational screen time, ≤ 2 h/d of recreational screen time had no significant association with better SRH

^d^ Compared to VPA every day, VPA 4–6 times a week, 2–3 times a week, once a week, once a month, less than once a month, or never was significantly positively associated with poorer SRH

^e^ Compared to ≥ 4 h/day of SB, < 4 h/day of SB was significantly positively associated with better SRH in girls and boys

^f^ Compared to > 2 h/d of recreational screen time and social media, ≤ 2h/d of recreational screen time and social media were significantly positively associated with better SRH

^g^ Compared to < 3 d/wk of VPA and muscle-strengthening activity, ≥ 3 d/wk of VPA and muscle-strengthening activity were significantly negatively associated with poorer SRH. Compared to < 5 d/wk of moderate PA, ≥ 5 d/wk of moderate PA had no significant association with poorer SRH

^h1^ Compared to ≥ 3 times/wk of PA, ≤ 2 times/wk of PA had no significant association with poorer SRH

^h2^ Compared to ≤ 2 h/d of TV, mobile phone email use and computer, > 2 h/d of TV, mobile phone email use and computer were not associated with poorer SRH in boys. In girls, 3–4 h/d of TV were significantly negatively associated with poorer SRH, and > 4 h/d mobile phone email use, and computer were significantly positively associated with poorer SRH in girls. Compared to 0 h/d of video games and telephones, > 0 h/d of video games and telephones were not associated with poorer SRH in boys. In girls, 0–1 h/d of video games was significantly positively associated with poorer SRH

^i^ Compared to ≤ 2 h/d of TV, > 2 h/d of TV had no significant association with poorer SRH on weekdays in boys and girls, but was positively associated with poorer SRH on weekends in boys, not girls. Compared to 0 h/d of video games/computer, 0.1 to 2.0 h/d of video games/computer were significantly negatively associated with poorer SRH on weekdays and weekends in girls. 2.1 h/d to 4 h/d of video games/computers were significantly negatively associated with poorer SRH in girls on weekdays, not weekends. > 4.0 of video games/computers were not significantly associated with poorer SRH on weekdays and weekends. In boys, > 0 h/d of video games/computer was not significantly associated with poorer SRH on weekdays and weekends

^j^ Compared to ≥ 2 h/d of TV, < 2 h/d of TV was significantly positively associated with better SRH. Compared to ≥ 2 h/d of computers, < 2 h/d of computers had no significant association with better SRH

^k^ Compared to ≥ 4 h/day of recreational screen time, < 4 h/day of recreational screen time had no significant association with better SRH

^l1^ out-of-school PA had no significant association with poorer SRH

^l2^ SB had no significant association with poorer SRH

^m^ Compared to active PA, insufficient PA had no association with poorer SRH, but inactive PA was significantly positively associated with poorer SRH

^n1^ Compared to ≥ 1 h/d of MVPA > 4 d/wk, ≤ 4 d/wk was significantly positively associated with poorer SRH

^n2^ Compared to < 4 h/d of TV, computer, and homework, ≥ 4 h/d of TV and homework were not significantly association with poorer SRH in girls and boys. ≥ 4 h/d of computers on weekdays was positively associated with poorer SRH in boys, not girls. ≥ 4 h/d of computers on weekends was not associated with poorer SRH in boys and girls

^o1^ Compared to active PA (≥ 3.0 kilocalories per kilogram per day), moderately (1.5–2.9 kilocalories per kilogram per day) and inactive PA (< 1.5 kilocalories per kilogram per day) were significantly positively associated with poorer SRH

^o2^ Compared to ≤ 2 h/d of recreational screen time, > 2 h/d of recreational screen time was significantly positively associated with poorer SRH

^p1^ Compared to ≥ 1 h/d of MVPA ≥ 5 d/wk, < 5 d/wk was significantly positively associated with poorer SRH

^p2^ Compared to < 2 h/d of TV, video games, and computers, > 2 h/d of TV, video games, and computers was significantly positively associated with poorer SRH

^q1^ Compared to ≥ 1 h/d of MVPA, < 1 h/d of MVPA was significantly positively associated with poorer SRH in boys, not girls

^q2^ Compared to ≤ 2 h/d of video games/computer and TV, > 2 h/d of video games/computer was significantly positively associated with poorer SRH in girls, not boys. > 2 h/d of TV was not significantly associated with poorer SRH in boys and girls. Compared to ≤ 1 h/d of reading and homework, > 1 h/d of reading was significantly negatively associated with poorer SRH in girls, not boys. > 1 h/d of homework was not significantly associated with poorer SRH in boys and girls. Compared to low tertiles of SB, the highest tertiles of SB were not significantly associated with poorer SRH in boys and girls

^r^ Compared to seldom PA (0 times a week, less than weekly or never), frequent PA (1 or more times a week or every day) was significantly negatively associated with poorer SRH

^s^ Compared to ≥ 3 d/wk of MVPA, < 3 d/wk of MVPA had no significant association with poorer SRH

^t1^ MVPA was significantly negatively associated with poorer SRH

^t2^ TV and Computer had no significant association with poorer SRH

^u^ Compared to MVPA (≥ 1 h/d, ≥ 6 d/wk), MVPA (≥ 1 h/d, < 6 d/wk) was significantly positively associated with poorer SRH in girls, not boys

^v1^ Compared to ≥ 1 d/wk of VPA and physical education, < 1d/wk of VPA and physical education had no significant association with poorer SRH. Compared to participants in team sports, no participant in team sports was significantly positively associated with poorer SRH in boys and girls

^v2^ Compared to < 1 h/d of TV, ≥ 1 h/d of TV had no significant association with poorer SRH in boys and girls

^w1^ Compared to MVPA (≥ 1 h/d, 7 d/wk), MVPA (≥ 1 h/d, < 7 d/wk) was significantly positively associated with poorer SRH in boys, not girls

^w2^ Compared to < 3 h/d of TV, ≥ 3 h/d of TV was significantly positively associated with poorer SRH in non-Hispanic white, but not in non-Hispanic black, and Hispanic. Compared to < 3 h/d of computers, ≥ 3 h/d of computers was significantly positively associated with poorer SRH in non-Hispanic white and Hispanic, but not non-Hispanic black

^x^ Compared to > 4 h/wk of VPA, 2–4 h/wk and < 2 h/wk of VPA were significantly positively associated with poorer SRH in boys, not girls

Among the 47 studies, none were rated as low quality (≤ 4 points), forty-two studies were classified as medium quality (5–9 points), and five were rated as high quality (≥ 10 points), with an average quality score of 7.19 points. Most studies were categorized as medium quality primarily due to their cross-sectional design, which cannot meet criteria such as measuring exposures (i.e. PA/SB) before outcomes (i.e. SRH), allowing a sufficient timeframe to observe potential associations, and assessing exposures multiple times over the study period. Additionally, most studies lacked information on sample size justification (*n* = 44), did not report whether outcome assessors were blinded to participants’ exposure status (*n* = 47), and whether the loss to follow-up remained within 20% after baseline (*n* = 46). The detailed quality ratings of the included studies are shown in Supplementary Fig. 1.

### Association of PA with SRH in children and adolescents

**Fig. 2:**
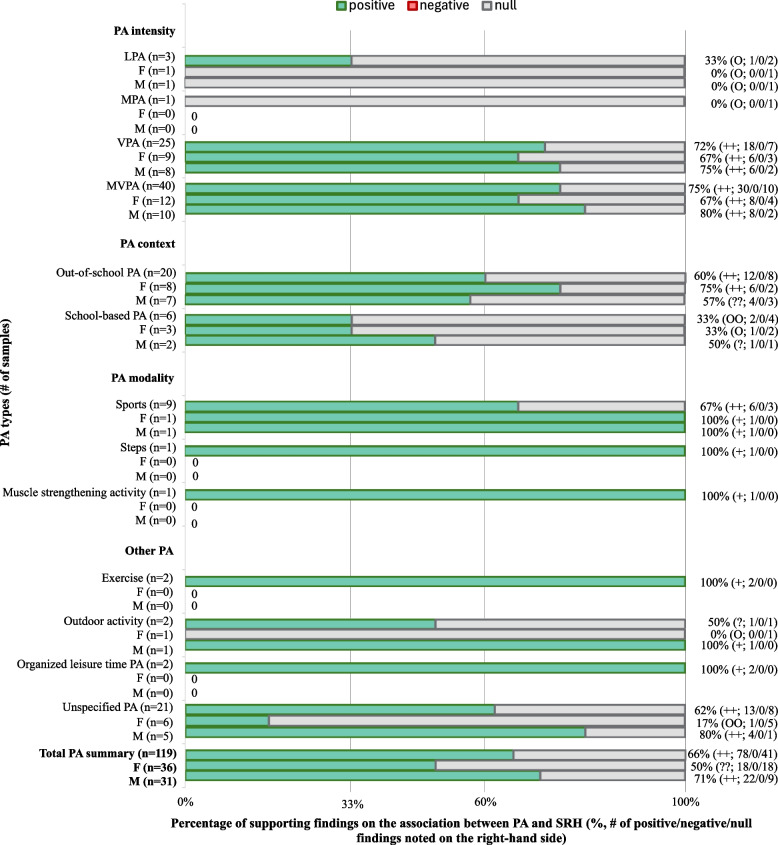
Percentage of supporting findings on the association between PA and SRH (%, # of positive/negative/null findings noted on the right-hand side). Notes: PA, physical activity; LPA, light physical activity; MPA, moderate physical activity; VPA, vigorous physical activity; MVPA, moderate to vigorous physical activity; M, male; F, female; 0–33%: no association; 34–59%: inconclusive; 60–100%: positive association. “++”, “– –”, “??”, or “OO” was coded when four or more samples supported one same direction associations. Not all studies reported findings between genders, and two only reported girls’ results [22, 47]. Fourteen samples represented out-of-school VPA, school-based organized sports, sports outside of school, or organized leisure time sports activity were only counted once in the final calculation of total PA to avoid double-counting, as they overlap in categories such as VPA, out-of-school PA, for detailed information please see Supplementary Table 1

Among the studies (*n* = 44) examining associations between PA and SRH, about 66% (*n* = 78) of the findings supported a positive association between PA and SRH in children and adolescents, and about 34% (*n* = 41) of them reported no association. The detailed percentage of PA intensity, type, context, and modality associated with SRH are summarized in Fig. 2, and detailed findings are summarized in Supplementary Table 1. Specifically, as seen in Fig. 2, VPA (72%; positive: *n* = 18, negative: *n* = 0, null: *n* = 7), MVPA (75%; positive: *n* = 30, negative: *n* = 0, null: *n* = 10), out-of-school PA (60%; positive: *n* = 12, negative: *n* = 0, null: *n* = 8), and sports (67%; positive: *n* = 6, negative: *n* = 0, null: *n* = 3) were double-coded as significantly positively associated with SRH. Further analysis of frequency and duration of PA revealed that ≥ 60 min/day of MVPA on ≥ 4 days/week (81%; positive: *n* = 22, negative: *n* = 0, null: *n* = 5), VPA on ≥ 3 days/week or ≥ 4 times/week (100%), out-of-school PA on ≥ 2 days/week (78%; positive: *n* = 7, negative: *n* = 0, null: *n* = 2) had the highest percentage of findings showing a significant positive association with SRH (Supplementary Table 4). Due to limited data, the association of sports participation frequency/duration with SRH remains unclear, with three findings suggesting no association for sports participation ≥ 1 time/week with SRH.

Among the 15 findings on the longitudinal association between PA and SRH (Supplementary Table 5), 40% (*n* = 6) indicate that baseline PA can predict SRH 2 to 6 years later. Of these, 75% of the VPA findings support a positive association with SRH (positive: *n* = 3, negative: *n* = 0, null: *n* = 1). However, the longitudinal association between PA and SRH were double-coded as inconclusive due to limited findings (40%) supporting a significant positive association.

### Association of SB with SRH in children and adolescents

**Fig. 3:**
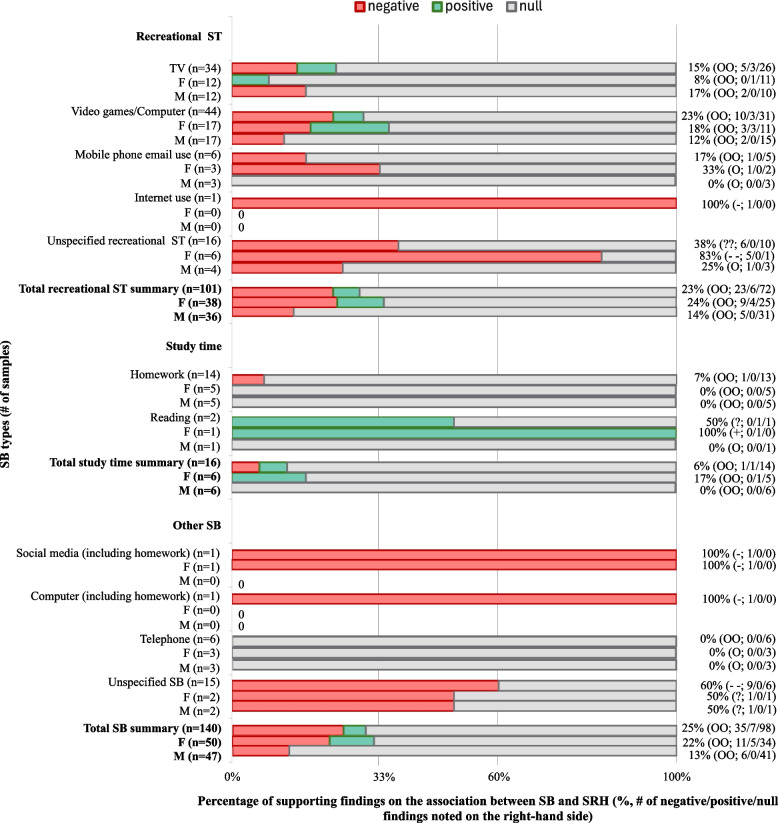
Percentage of supporting findings on the association between SB and SRH (%, # of negative/positive/null findings noted on the right-hand side). Notes: SB, sedentary behavior; ST, screen time; M, male; F, female. 0–33%: no association; 34–59%: inconclusive; 60–100%: negative or positive association. “++”, “– –”, “??”, or “OO” was coded when four or more samples supported one same direction associations. Not all studies reported findings between genders, and two only reported girls’ results [22, 47]. For detailed information please see Supplementary Table 2

Among the included studies (*n* = 26) that evaluated the association between SB and SRH, about 70% (*n* = 98) of the findings show no association, 25% (*n* = 35) show negative association, and about 5% (*n* = 7) show positive association. As seen in Fig. 3, most types of SB show no significant association with SRH. Negative associations with SRH were reported in 23% of findings for total recreational screen time (negative: *n* = 23, positive: *n* = 6, null: *n* = 72), 15% for TV watching (negative: *n* = 5, positive: *n* = 3, null: *n* = 26), 23% for video game/computer use (negative: *n* = 10, positive: *n* = 3, null: *n* = 31), and 17% for mobile phone email use (negative: *n* = 1, positive: *n* = 0, null: *n* = 5). In contrast, negative associations with SRH were less frequently reported for SB, such as total study time (6%; negative: *n* = 1, positive: *n* = 1, null: n = 14), homework time (7%; negative: *n* = 1, positive: *n* = 0, null: *n* = 13), and telephone use (0%; negative: *n* = 0, positive: *n* = 0, null: *n* = 6). A detailed summary of SB and SRH findings is available in Supplementary Table 2.

Three findings examined the longitudinal association between recreational screen time and SRH, all indicating no significant association between recreational screen time and SRH (Supplementary Table 5).

### Association of PA and SB with SRH in children and adolescents of different genders

Evidence suggests that the percentage of findings on the association of PA with SRH varied between boys and girls. Overall, findings of PA positively associated with SRH were high (71%; positive: *n* = 22, negative: *n* = 0, null: *n* = 9) in boys, while overall findings of PA association with girls' SRH were inconclusive (50%; positive: *n* = 18, negative: *n* = 0, null: *n* = 18). Specifically, as shown in Fig. 2, MVPA and VPA were double-coded as significantly positively associated with SRH in both boys and girls. Among boys, 80% of MVPA findings (*n* = 8) and 75% of VPA findings (*n* = 6) indicated positive associations, with no negative findings and two null results (MVPA: *n* = 2; VPA: *n* = 2). Similarly, among girls, 67% of MVPA findings (*n* = 8) and 67% of VPA findings (*n* = 6) demonstrated positive associations, with no negative findings and few null results (MVPA: *n* = 4; VPA: *n* = 3) across the included studies. Out-of-school PA was double-coded as positively associated with girls' SRH (75%; positive: *n* = 6, negative: *n* = 0, null: *n* = 2), while the association for boys was double-coded as inconclusive (57%; positive: *n* = 4, negative: *n* = 0, null: *n* = 3). As shown in Fig. 3, most findings showed that SB types were not significantly associated with SRH. However, the percentage of findings reporting negative associations between SB and SRH is higher in girls (22%; negative: *n* = 11, positive: *n* = 5, null: *n* = 34) compared to boys (13%; negative: *n* = 6, positive: *n* = 0, null: *n* = 41).

Longitudinal studies suggest that PA predicts later SRH in boys (71%; positive: *n* = 5, negative: *n* = 0, null: *n* = 2) but not in girls (14%; positive: *n* = 1, negative: *n* = 0, null: *n* = 6). Due to limited findings, a longitudinal conclusion on SB and SRH across genders cannot be drawn (Supplementary Table 5).

## Discussion

This systematic review aimed to summarize and analyze studies on the association of PA and SB with SRH in children and adolescents. Of the included studies, we found that ≥ 60 min/day of MVPA (≥ 4 days/week), VPA (≥ 3 days/week or ≥ 4 times/week), out-of-school PA (≥ 2 days/week), and sports participation are significantly positively associated with SRH among children and adolescents. The majority of research findings suggest that SB, including TV watching, video games/computer use, mobile phone email use, homework, and telephone had no significant association with SRH. Regarding gender differences, more research findings supported positive PA association with SRH in boys than in girls (71% vs. 50%). While SB showed no overall association, it was more frequently reported as negatively association with SRH in girls than in boys (22% vs. 13%).

### Association of PA with SRH in children and adolescents

The positive impact of regular PA on health is widely recognized by scholars. This review adds to the previous review’s findings [21], highlighting the crucial role of PA in improving the SRH of children and adolescents. However, the longitudinal association between PA and SRH remains inconclusive, as these studies examine the association of baseline PA on later SRH (2–6 years), a relationship that could be influenced by changes in PA behavior throughout this developmental period [68]. The examination of findings on various intensities of PA reveals that VPA and MVPA exhibit a significantly positive association with SRH in children and adolescents. This may be due to the health benefits of VPA and MVPA, such as reducing depression and combating obesity in this demographic [69]. Additionally, longitudinal findings indicate a positive association between VPA and SRH in children and adolescents, suggesting that high initial engagement in VPA may support sustained or increased PA over time [68], thereby maintaining or even enhancing SRH. Alternatively, these findings could reflect the lasting positive effects of early VPA engagement on health perceptions and behaviors over the developmental period. Furthermore, our analysis reveals that 79% of findings from included studies reporting at least 60 min of daily MVPA were positively associated with SRH. This supports the World Health Organization’s PA guidelines for improving children’s and adolescent’s health [19]. Additionally, the findings indicate that engaging in ≥ 60 min of MVPA, even as little as once per week, can be positively associated with SRH, suggesting that even with a lower frequency of ≥ 60 min of MVPA than the recommended daily amount of 60 min could potentially be beneficial for children and adolescents.

There is inadequate evidence suggesting that school-based PA had a significant association with SRH in children and adolescents, potentially due to variations in the frequency of physical education classes across studies, the primary component of school-based PA. For example, Zullig et al. (2011) found no significant difference in SRH between students who participated in physical education classes at least once a week and those who did not participate at all [62]. Conversely, Jodkowska et al. (2019) reported that students with nearly full attendance in physical education classes had better SRH compared to their peers [22]. It is possible that these contrasting findings may result from the different durations and frequencies of PA participation, which will likely determine its impact on SRH. Additionally, lower attendance may indicate absences due to illness, potentially leading to lower SRH reports.

Findings in this review show that out-of-school PA participation is positively associated with SRH in children and adolescents. The variety of sports programs and the voluntary nature of participation in out-of-school activities distinguish them from structured school activities. Research suggests that active involvement in out-of-school PA fosters higher self-esteem and lower rates of depression and provides valuable opportunities for growth and development among children and adolescents [70]. Moreover, findings suggest that participating in out-of-school PA at least two days per week is significantly positively associated with SRH. Notably, most of these ≥ 2 days/week activities involve vigorous intensity, highlighting the importance of high-intensity PA in out-of-school settings.

### Association of SB with SRH in children and adolescents

In this systematic review, findings from included studies demonstrated that SB was not significantly associated with SRH in children and adolescents. This contrasts with previous review findings that identified a negative association between SB and SRH (odds ratio = 1.28, 95% CI = 1.20–1.36) through meta-analysis five years ago [21]. This discrepancy may be attributed to differences in methodology. For example, our review employed a semi-quantitative review method [36] to code and summarize the studies, while Zhang et al. [21] mainly utilized a meta-analysis approach. Second, this review included 18 additional studies published in the last five years, potentially reflecting findings shift in recent years. Third, while this review focused on those aged 5 to 17 years old, the earlier review included children and adolescents aged 3 to 19 [21]. It is important to note that children and adolescents generally rate their health as good [71], and tend to have better SRH than adults [72]. Although evidence in this review suggests no significant association between total SB and SRH among young children and adolescents, early signs of health issues in this demographic could signal potential future challenges. Given that childhood and adolescence are critical periods to prevent health-risk behavior [73], implementing strategies during this period to reduce prolonged SB may be necessary to prevent potential future negative effects on their health.

Findings suggest that total recreational screen time had no association with SRH in children and adolescents. Different types of screen time may have distinct effects on SRH in this population. Research indicates that most screen time for children and adolescents is spent on mobile phones and tablets [74]. The limited focus on these devices in the existing studies warrants further investigation to arrive at definitive conclusions about their association with SRH. Similarly, findings show that study time had no significant association with SRH. This may be partly explained by the fact that homework, the major component in this category, is believed to enhance academic performance [75] and might potentially alleviate the stress of academic performance. Nonetheless, more research is needed to examine the associations between different SB and SRH and potential confounding factors that may influence them.

### Association of PA and SB with SRH in children and adolescents of different genders

Findings from the included studies show variation in the association of PA and SB with SRH of children and adolescents between genders. While 71% of findings from included studies indicated a positive association between PA and SRH among boys, that was about 50% for girls. SB showed no association with SRH in both genders; however, 13% of findings from included studies show a negative association of SB with SRH among boys, compared to 22% in girls. This discrepancy may be influenced by gender differences in health perceptions, as girls generally report lower SRH than boys [66]. Biological and psychological changes during puberty could also play a role. For instance, girls typically experience an increase in body fat during puberty, which can negatively affect their SRH [14], while boys tend to experience a decrease in body fat during this period [76]. Additionally, depression rates rose more significantly in girls during this period compared to boys [77], which may further impact their SRH [5].

To address these disparities, gender-specific PA interventions are needed. For girls, our review suggests that out-of-school PA had the highest percentage of findings supporting a positive association with SRH. Beyond its previously noted mental health benefits, such as reducing depression [70], this association may be partly explained by girls engaging in significantly more MVPA outside of school than during school hours [78]. Given that girls’ MVPA also showed a positive association with SRH in our review, interventions should prioritize increasing their participation in out-of-school MVPA to enhance their SRH. For boys, MVPA had the highest percentage of findings supporting a positive association with SRH, suggesting that moderate- to high-intensity activities may be the most effective strategy for promoting SRH in boys.

### Future directions

Future research should explore a wider range of PA modalities and contexts to assess their association with SRH in children and adolescents. While some studies examined specific modalities of PA, such as step count and muscle-strengthening exercises [8, 12], the limited number of studies on these modalities constrains our ability to draw conclusions. Second, future studies should use consistent terminology to clearly reflect the intensity, context, and modalities of PA measured, as many current studies often lack clarity. For example, some studies asked about PA, which causes participants to sweat or be out of breath, but did not label it as VPA (see supplementary Table 3). Using consistent terminology will enhance researchers' ability to evaluate how different PA intensities, modalities, and contexts impact SRH. It will also provide more precise guidance for designing health interventions and ensure that critical information isn't overlooked. The same applies to studies on SB. Third, studies on more prominent daily screen time (mobile phones and tablets) [74] are recommended to reflect current trends better and assess their association with SRH. Most of the included studies used "screen time" as a broad term, covering recreational, non-recreational use, or both, often without clarification. Future research should distinguish between recreational screen time and non-recreational screen time, as they may yield varying outcomes. Additionally, the use of screens for homework/study has increased among children and adolescents. Examining the impact of screen-based versus non-screen-based homework/study time on SRH could yield valuable insights as well.

### Limitations

This review presents several limitations that warrant consideration. The search was limited to three major databases, potentially overlooking relevant articles from other sources that were not indexed in those databases. Additionally, the review included articles written in English only, which may have excluded important research published in other languages, leading to potential bias in the findings. Furthermore, we used a semi-quantitative review method to synthesize findings, rather than a meta-analysis. While this method allowed us to summarize diverse studies systematically, it does not provide a quantitative measure of effect size, limiting our ability to assess the strength of observed associations comprehensively. Moreover, this review was not pre-registered, which may affect transparency and reproducibility in the study selection and synthesis process. Finally, a potential limitation lies in the high prevalence of cross-sectional designs used among the included studies. As a result, the conclusions drawn from this review may not infer causal relationships between PA, SB, and SRH in children and adolescents. Additionally, while our review aimed to explore these associations, we acknowledge the potential for reverse causation, where SRH could also influence PA and SB.

## Conclusions

This systematic review synthesized findings from 47 studies on the association of PA and SB with SRH in children and adolescents. The evidence suggests that engaging in ≥ 60 min/day of MVPA on ≥ 4 days/week, VPA on ≥ 3 days/week or ≥ 4 times/week, out-of-school VPA on ≥ 2 days/week, and sports participation was significantly positively associated with SRH. Notably, even lower frequency participation in high-intensity PA (MVPA, VPA) than recommended daily participation was positively associated with SRH. Additionally, more evidence supported a positive association between PA and SRH in boys than girls, while more evidence supported a negative association between SB and SRH in girls than boys. It suggests that health intervention design should consider these gender differences and the challenges girls face during childhood and adolescence [76, 77].

## Supplementary Information

Supplementary Material 1.

## Data Availability

No datasets were generated or analysed during the current study.

## Abbreviations

PA: Physical activity
SB: Sedentary behavior
SRH: Self-rated health
MVPA: Moderate to vigorous physical activity
VPA: Vigorous physical activity
