# Supplementary material for: Associations of different types of physical activity and sedentary behavior with self-rated health in children and adolescents: a systematic review of research from 2010 to 2024
Source: Int J Behav Nutr Phys Act. 2025 Apr 21;22:48. doi: 10.1186/s12966-025-01747-2 (PMC12013060; doi:10.1186/s12966-025-01747-2)
Supplement: Supplementary file 1 — Supplementary Material 1. [file 12966_2025_1747_MOESM1_ESM.docx]

| Supplementary Table 1. Summary of the association of physical activity with self-rated health among children and adolescents | | | | | | | | | | | | | | |
| --- | --- | --- | --- | --- | --- | --- | --- | --- | --- | --- | --- | --- | --- | --- |
| PA | Literatures | Association | Summary | | | | | | | | | | | |
|  |  |  | All | | | | Boys | | | | Girls | | | |
|  |  |  | # of subgroup | + | - | O | # of subgroup | + | - | O | # of subgroup | + | - | O |
| **PA Intensity** |  |  |  |  |  |  |  |  |  |  |  |  |  |  |
| LPA | (Husu et al.,2016) | + | 3 | 1 | 0 | 2 | 1 | 0 | 0 | 1 | 1 | 0 | 0 | 1 |
|  | (Herman et al.,2014)M; (Herman et al.,2014)F | O |  |  |  |  |  |  |  |  |  |  |  |  |
| MPA | (Hwang et al.,2018)^(≥ 5 d/wk)^ | O | 1 | 0 | 0 | 1 | 0 | 0 | 0 | 0 | 0 | 0 | 0 | 0 |
| VPA | (Jodkowska et al.,2019)F^(≥ 2 d/wk) out-of-school VPA^; (Hwang et al.,2018)^(≥ 3 d/wk)^; (Lachytova et al.,2017)^(4–6 times/wk)^; (Lachytova et al.,2017)^(everyday)^; (Jerdén et al.,2011)M^(> 3times/wk) out-of-school VPA^; (Jerdén et al.,2011)F^(> 3times/wk) out-of-school VPA^; (Herman et al.,2014)M; (Pierannunzio et al.,2022)F^(≥ 2 d/wk) out-of-school VPA^ ^in 11 year old^; (Pierannunzio et al.,2022)F^(≥ 2 d/wk) out-of-school VPA^ ^in 13 year old^; (Pierannunzio et al.,2022)F^(≥ 2 d/wk) out-of-school VPA^ ^in 15 year old^; (Pierannunzio et al.,2022)M^(≥ 2 d/wk) out-of-school VPA^ ^in 13 year old^; (Pierannunzio et al.,2022)M^(≥ 2 d/wk) out-of-school VPA^ ^in 15 year old^; (Wang et al.,2022)^(7 times/wk)^; (Liang et al., 2024)M^(> 1 h/d)^; (Liang et al., 2024)F^(> 1 h/d)^; (Spein et al.,2013)^(≥ 1 times/wk) out-of-school VPA in Sami^; (Spein et al.,2013)^(everyday) in Inuit^; (Elinder et al.,2011)M^(> 4 h/wk)^ | + | 25 | 18 | 0 | 7 | 8 | 6 | 0 | 2 | 9 | 6 | 0 | 3 |
|  | (Pierannunzio et al.,2022)M^(≥ 2 d/wk) out-of-school VPA^ ^in 11 year old^; (Zullig et al.,2011)M^(≥1d/wk)^; (Zullig et al.,2011)F^(≥1d/wk)^; (Herman et al.,2014)F; (Lachytova et al.,2017)^(1 times/wk)^; (Lachytova et al.,2017)^(2–3 times/wk)^; (Elinder et al.,2011)F^(> 4 h/wk)^ | O |  |  |  |  |  |  |  |  |  |  |  |  |
| MVPA | (Herman et al.,2014)M^(≥ 1 h/d)^; (Jodkowska et al.,2019)F^(≥ 1 h/d)^; (Foti et al.,2010)M^(≥ 1 h/d^); (Granger et al.,2017)^(≥ 1 h/d)^; (de Fátima Guimarães et al.,2022)F^(≥ 1 h/d)^; (Richter et al.,2012)F^(≥ 1 h/d, ≥ 6 d/wk)^; (Martinez-Lopez et al.,2015)M^(≥ 1 h/d, > 4 d/wk)^; (Martinez-Lopez et al.,2015)F^(≥ 1 h/d, > 4 d/wk)^; (Tabak et al.,2012)^Rural^; (Tabak et al.,2012)^urban^; (Moor et al.,2014)^(≥ 1 h/d, ≥ 5 d/wk)^; (Pierannunzio et al.,2022)M^(≥ 1 h/d, ≥ 4 d/wk) 11 years old^; (Pierannunzio et al.,2022)F^(≥ 1 h/d, ≥ 4 d/wk) 13 years old^; (Pierannunzio et al.,2022)M^(≥ 1 h/d, ≥ 4 d/wk) 13 years old^; (Pierannunzio et al.,2022)F^(≥ 1 h/d, ≥ 4 d/wk) 15 years old^; (Pierannunzio et al.,2022)M^(≥ 1 h/d, ≥ 4 d/wk) 15 years old^; (Wang et al.,2023); (Kyan et al.,2022)^(≥ 1 h/d) junior high school students^; (Karchynskaya et al.,2022)^(≥ 1 h/d, 7 d/wk)^; (Karchynskaya et al.,2022)^(≥ 1 h/d, 5-7 d/wk)^; (Moran et al., 2024)^(≥ 1 h/d, 1-2 d/wk)^; (Moran et al., 2024)^(≥ 1 h/d, 3-4 d/wk)^; (Moran et al., 2024)^(≥ 1 h/d, 5-6 d/wk)^; (Moran et al., 2024)^(≥ 1 h/d, 7 d/wk)^; (Liang et al., 2024)M^(≥ 1 h/d) weekdays^; (Liang et al., 2024)F^(≥ 1 h/d) weekdays^;(Liang et al., 2024)M^(≥ 1 h/d) weekends^; (Liang et al., 2024)F^(≥ 1 h/d) weekends^; (de Sales et al., 2024); (Shi et al., 2023)^(≥ 1 h/d)^ | + | 40 | 30 | 0 | 10 | 10 | 8 | 0 | 2 | 12 | 8 | 0 | 4 |
|  | (Herman et al.,2014)F^(≥ 1 h/d)^; (Richter et al.,2012)M^(≥ 1 h/d, ≥ 6 d/wk)^; (Husu et al.,2016); (Nigg et al.,2015); (Afridi et al.,2013)^(≥ 3 d/wk)^; (Foti et al.,2010)F^(≥ 1 h/d)^; (Joensuu et al., 2024)M; (Joensuu et al., 2024)F; (Kyan et al.,2022)^(≥ 1 h/d) elementary school students^; (Pierannunzio et al.,2022)F^(≥ 1 h/d, ≥ 4 d/wk) 11 years old^ | O |  |  |  |  |  |  |  |  |  |  |  |  |
| **PA context** |  |  |  |  |  |  |  |  |  |  |  |  |  |  |
| School-based PA | (Jodkowska et al.,2019)F^(attend all and almost all classes)^^; (Liang et al., 2024)M^(> 1 h/d)^ | + | 6 | 2 | 0 | 4 | 2 | 1 | 0 | 1 | 3 | 1 | 0 | 2 |
|  | (Zullig et al.,2011)M^(≥1d/wk)^ ^; (Zullig et al.,2011)F^(≥1d/wk)^ ^; (Curtin et al.,2018)^(≥ 1 times/wk) school-based organized sports^; (Liang et al., 2024)F^(> 1 h/d)^ | O |  |  |  |  |  |  |  |  |  |  |  |  |
| Out-of-school PA | (Jodkowska et al.,2019)F^(≥ 2 d/wk) out-of-school VPA^; (Jerdén et al.,2011)M^(> 3times/wk) out-of-school VPA^; (Jerdén et al.,2011)F^(> 3times/wk) out-of-school VPA^; (Spein et al.,2013)^(≥ 1 times/wk) out-of-school VPA in Sami^; (Kantomaa et al.,2015)F^the^ ^highest tertile metabolic equivalent of task h/wk^; (Kantomaa et al.,2015)M^the highest tertile metabolic equivalent of task^ ^h/wk^; (Pierannunzio et al.,2022)F^(≥ 2 d/wk) out-of-school VPA^ ^in 11 year old^; (Pierannunzio et al.,2022)F^(≥ 2 d/wk) out-of-school VPA^ ^in 13 year old^; (Pierannunzio et al.,2022)F^(≥ 2 d/wk) out-of-school VPA^ ^in 15 year old^; (Pierannunzio et al.,2022)M^(≥ 2 d/wk) out-of-school VPA^ ^in 13 year old^; (Pierannunzio et al.,2022)M^(≥ 2 d/wk) out-of-school VPA^ ^in 15 year old^; (Yang et al., 2023)^(> 1 h/d) Housework^ | + | 20 | 12 | 0 | 8 | 7 | 4 | 0 | 3 | 8 | 6 | 0 | 2 |
|  | (Pierannunzio et al.,2022)M^(≥ 2 d/wk) out-of-school VPA^ ^in 11 year old^; (Yang et al., 2023)^(0.5–1 h/d) Housework^; (Curtin et al.,2018)^(≥ 1 times/wk) sport outside of school^; (Smith et al.,2015); (Kantomaa et al.,2015)F^the^ ^middle tertile metabolic equivalent of task h/wk^; (Kantomaa et al.,2015)M^the^ ^middle tertile metabolic equivalent of task h/wk^; (Jerdén et al.,2011)F^(> 3times/wk)^; (Jerdén et al.,2011)M^(> 3times/wk)^ | O |  |  |  |  |  |  |  |  |  |  |  |  |
| **PA Modality** |  |  |  |  |  |  |  |  |  |  |  |  |  |  |
| Muscle strengthening activity | (Hwang et al., 2018)^(≥ 3 d/wk)^ | + | 1 | 1 | 0 | 0 | 0 | 0 | 0 | 0 | 0 | 0 | 0 | 0 |
| Steps | (Husu et al.,2016) | + | 1 | 1 | 0 | 0 | 0 | 0 | 0 | 0 | 0 | 0 | 0 | 0 |
| Sports | (Badura et al.,2021)^organized leisure time sports activity^; (Zullig et al.,2011)M; (Zullig et al.,2011)F; (Badura et al., 2015)^individual sports^; (Badura et al., 2015)^team sports^; (Karchynskaya et al.,2022)^organized leisure time sports activity^ | + | 9 | 6 | 0 | 3 | 1 | 1 | 0 | 0 | 1 | 1 | 0 | 0 |
|  | (Curtin et al.,2018)^(≥ 1 times/wk)^ ^after school/Saturday school-based organized sport^; (Curtin et al.,2018)^(≥ 1 times/wk) school-based organized sports^; (Curtin et al.,2018)^(≥ 1 times/wk)^ ^sport outside of school^ | O |  |  |  |  |  |  |  |  |  |  |  |  |
| **Other PA** |  |  |  |  |  |  |  |  |  |  |  |  |  |  |
| Unspecified PA | (Park, 2024)^2019^; (Park, 2024)^2020^; (Park, 2024)^2021^; (Gonzalez-Alvarez et al., 2023)^5–6 times/wk^; (Gonzalez-Alvarez et al., 2023)^≥ 7 times/wk^; (Joensuu et al., 2024)M; (Tebar et al.,2021)M^(≥ 300 min/wk)^; (Moral-García et al.,2020); (Marques et al.,2019)^(≥ 1 h/d)^; (Meireles et al.,2015)^Active PA vs inactive^; (Herman et al.,2015)F^≥ 3.0 kilocalories per kilogram per day^; (Herman et al.,2015)M^≥ 3.0 kilocalories per kilogram per day^; (Spengler et al.,2014)M | + | 21 | 13 | 0 | 8 | 5 | 4 | 0 | 1 | 6 | 1 | 0 | 5 |
|  | (Gonzalez-Alvarez et al., 2023)^1-2 times/wk^; (Gonzalez-Alvarez et al., 2023)^3-4 times/wk^; (Joensuu et al., 2024)F; (Tebar et al.,2021)F^(≥ 300 min/wk)^; (Li et al.,2018)F^(≥ 3 times/wk)^; (Li et al.,2018)M^(≥ 3 times/wk)^; (Spengler et al.,2014)F; (Meireles et al.,2015)^Active PA vs insufficiently PA^ | O |  |  |  |  |  |  |  |  |  |  |  |  |
| Organized leisure time PA | (Karchynskaya et al.,2022)^organized leisure time sports activity^; (Badura et al.,2021)^organized leisure time sports activity^ | + | 2 | 2 | 0 | 0 | 0 | 0 | 0 | 0 | 0 | 0 | 0 | 0 |
| Outdoor activity | (Liu et al.,2015)M | + | 2 | 1 | 0 | 1 | 1 | 1 | 0 | 0 | 1 | 0 | 0 | 1 |
|  | (Liu et al.,2015)F | O |  |  |  |  |  |  |  |  |  |  |  |  |
| Exercise | (Yang et al., 2023)^(0.25–0.5 h/d)^; (Yang et al., 2023)^(> 0.5 h/d)^ | + | 2 | 2 | 0 | 0 | 0 | 0 | 0 | 0 | 0 | 0 | 0 | 0 |
| Total PA summary |  |  | 119 | 78 | 0 | 41 | 31 | 22 | 0 | 9 | 36 | 18 | 0 | 18 |

Notes. PA: physical activity; LPA: Light physical activity; MPA: Moderate physical activity; VPA: Vigorous physical activity; MVPA: Moderate to vigorous physical activity; M: Male; F: Female; ^: physical education; O: No significant association; +; Significant positive association; -: Significant negative association.

(Jodkowska et al.,2019)F^(≥ 2 d/wk) out-of-school VPA^; (Jerdén et al.,2011)M^(> 3times/wk) out-of-school VPA^; (Jerdén et al.,2011)F^(> 3times/wk) out-of-school VPA^; (Spein et al.,2013)^(≥ 1 times/wk) out-of-school VPA in Sami^; (Pierannunzio et al.,2022)F^(≥ 2 d/wk) out-of-school VPA^ ^in 11 year old^; (Pierannunzio et al.,2022)F^(≥ 2 d/wk) out-of-school VPA^ ^in 13 year old^; (Pierannunzio et al.,2022)F^(≥ 2 d/wk) out-of-school VPA^ ^in 15 year old^; (Pierannunzio et al.,2022)M^(≥ 2 d/wk) out-of-school VPA^ ^in 13 year old^; (Pierannunzio et al.,2022)M^(≥ 2 d/wk) out-of-school VPA^ ^in 15 year old^; (Pierannunzio et al.,2022)M^(≥ 2 d/wk) out-of-school VPA^ ^in 11 year old^; (Curtin et al.,2018)^(≥ 1 times/wk) school-based organized sports^; (Curtin et al.,2018)^(≥ 1 times/wk)^ ^sport outside of school^; (Badura et al.,2021)^organized leisure time sports activity^; (Karchynskaya et al.,2022)^organized leisure time sports activity^. These instances were only counted once in the final calculation of total PA to avoid double-counting, as they overlap in categories such as VPA, out-of-school PA, sport, school-based PA, and organized leisure time PA.

| Supplementary Table 2. Summary of the association of sedentary behavior with self-rated health among children and adolescents | | | | | | | | | | | | | | |
| --- | --- | --- | --- | --- | --- | --- | --- | --- | --- | --- | --- | --- | --- | --- |
| SB | Literatures | Association | Summary | | | | | | | | | | | |
|  |  |  | All | | | | Boys | | | | Girls | | | |
|  |  |  | # of subgroup | + | - | O | # of subgroup | + | - | O | # of subgroup | + | - | O |
| **Recreational ST** |  |  |  |  |  |  |  |  |  |  |  |  |  |  |
| TV | (Moor et al.,2014)^(> 2 h/d)^; (Lachytova et al.,2017)^(≥ 2 h/d)^; (Silva et al.,2017)M^(2.1-4.0 h/d) weekend^; (Silva et al.,2017)M^(> 4.0 h/d) weekend^; (Foti et al.,2010)^(≥3 h/d) Non-Hispanic white^ | - | 34 | 3 | 5 | 26 | 12 | 0 | 2 | 10 | 12 | 1 | 0 | 11 |
|  | (Yang et al., 2023)^(1–2 h/d)^; (Yang et al., 2023)^(> 2 h/d)^; (Li et al.,2018)F^(3–4 h/d)^ | + |  |  |  |  |  |  |  |  |  |  |  |  |
|  | (Li et al.,2018)M^(2–3 h/d)^; (Li et al.,2018)M^(3–4 h/d)^; (Li et al.,2018)M^(> 4 h/d)^; (Li et al.,2018)F^(2–3 h/d)^; (Li et al.,2018)F^(> 4 h/d)^; (Herman et al.,2014)M^(> 2 h/d)^; (Herman et al.,2014)F^(> 2 h/d)^; (Zullig et al.,2011)M^(≥1 h/d)^; (Zullig et al.,2011)F^(≥1 h/d)^; (Martinez-Lopez et al.,2015)M^(≥ 4 h/d) weekdays^; (Martinez-Lopez et al.,2015)F^(≥ 4 h/d) weekdays^; (Martinez-Lopez et al.,2015)M^(≥ 4 h/d) weekend^; (Martinez-Lopez et al.,2015)F^(≥ 4 h/d) weekend^; (Tabak et al.,2012)^Rural^; (Tabak et al.,2012)^urban^; (Meireles et al., 2015); (Werneck et al.,2018)M; (Werneck et al.,2018)F; (Silva et al.,2017)M^(2.1-4.0 h/d) weekdays^; (Silva et al.,2017)M^(> 4.0 h/d) weekdays^; (Silva et al.,2017)F^(2.1-4.0 h/d) weekdays^; (Silva et al.,2017)F^(> 4.0 h/d) weekdays^; (Silva et al.,2017)F^(2.1-4.0 h/d) weekend^; (Silva et al.,2017)F^(> 4.0 h/d) weekend^; (Foti et al.,2010)^(≥3 h/d) Non-Hispanic black^; (Foti et al.,2010)^(≥3 h/d) Hispanic^ | O |  |  |  |  |  |  |  |  |  |  |  |  |
| Video games/Computer | (Martinez-Lopez et al.,2015)M^(≥ 4 h/d) weekdays^; (Herman et al.,2014)F^(> 2 h/d)^; (Moor et al.,2014)^(> 2 h/d) video game^; (Yang et al., 2023) ^(1–2 h/d)^; (Yang et al., 2023) ^(> 2 h/d)^; (Werneck et al.,2018)M; (Li et al.,2018)F^(0–1 h/d) video games^; (Li et al.,2018)F^(> 4 h/d) computer^; (Foti et al.,2010)^(≥3 h/d) Non-Hispanic white^; (Foti et al.,2010)^(≥3 h/d) Hispanic^ | - | 44 | 3 | 10 | 31 | 17 | 0 | 2 | 15 | 17 | 3 | 3 | 11 |
|  | (Silva et al.,2017)F^(0.1-2.0 h/d) weekdays^; (Silva et al.,2017)F^(2.1-4.0 h/d) weekdays^; (Silva et al.,2017)F^(0.1-2.0 h/d) weekend^ | + |  |  |  |  |  |  |  |  |  |  |  |  |
|  | (Silva et al.,2017)M^(0.1-2.0 h/d) weekdays^; (Silva et al.,2017)M^(2.1-4.0 h/d) weekdays^; (Silva et al.,2017)M^(> 4.0 h/d) weekdays^; (Silva et al.,2017)M^(0.1-2.0 h/d) weekend^; (Silva et al.,2017)M^(2.1-4.0 h/d) weekend^; (Silva et al.,2017)M^(> 4.0 h/d) weekend^; (Silva et al.,2017)F^(> 4.0 h/d) weekdays^; (Silva et al.,2017)F^(2.1-4.0 h/d) weekend^; (Silva et al.,2017)F^(> 4.0 h/d) weekend^; (Li et al.,2018)F^(1–2 h/d) video games^; (Li et al.,2018)F^(2–3 h/d) video games^; (Li et al.,2018)F^(> 3 h/d) video games^; (Li et al.,2018)M^(0–1 h/d) video games^; (Li et al.,2018)M^(1–2 h/d) video games^; (Li et al.,2018)M^(2–3 h/d) video games^; (Li et al.,2018)M^(> 3 h/d) video games^; (Martinez-Lopez et al.,2015)M^(≥ 4 h/d) weekend^;(Martinez-Lopez et al.,2015)F^(≥ 4 h/d) weekdays^;(Martinez-Lopez et al.,2015)F^(≥ 4 h/d) weekend^; (Li et al.,2018)M^(2–3 h/d) computer^; (Li et al.,2018)M^(3–4 h/d) computer^; (Li et al.,2018)M^(> 4 h/d) computer^; (Li et al.,2018)F^(2–3 h/d) computer^; (Li et al.,2018)F^(3–4 h/d) computer^; (Herman et al.,2014)M^(> 2 h/d)^; (Lachytova et al.,2017)^(≥ 2 h/d)^; (Tabak et al.,2012)^Rural^; (Tabak et al.,2012)^urban^; (Meireles et al., 2015); (Werneck et al.,2018)F; (Foti et al.,2010)^(≥3 h/d) Non-Hispanic black^ | O |  |  |  |  |  |  |  |  |  |  |  |  |
| Mobile phone email use | (Li et al.,2018)F^(> 4 h/d)^ | - | 6 | 0 | 1 | 5 | 3 | 0 | 0 | 3 | 3 | 0 | 1 | 2 |
|  | (Li et al.,2018)M^(2–3 h/d)^; (Li et al.,2018)M^(3–4 h/d)^; (Li et al.,2018)M^(> 4 h/d)^; Li et al.,2018)F^(2–3 h/d)^; (Li et al.,2018)F^(3–4 h/d)^ | O |  |  |  |  |  |  |  |  |  |  |  |  |
| Internet use | (Du et al., 2024) | - | 1 | 0 | 1 | 0 | 0 | 0 | 0 | 0 | 0 | 0 | 0 | 0 |
| Unspecified recreational ST | (Herman et al.,2015)M^(> 2 h/d) †^; (Herman et al.,2015)F^(> 2 h/d) †^; (Liang et al., 2024)F^(> 2 h/d) weekdays^; (Liang et al., 2024)F^(> 2 h/d) weekends^; (de Fátima Guimarães et al., 2022)F^(> 2h/d)†^; (Jodkowska et al.,2019)F^(> 2 h/d) †^ | - | 16 | 0 | 6 | 10 | 4 | 0 | 1 | 3 | 6 | 0 | 5 | 1 |
|  | (Marques et al.,2019)^(≥ 2 h/d)^; (Kyan et al.,2022)^(> 2 h/d)^  ^junior high school students†^; (Kyan et al.,2022)^(> 2 h/d) elementary school students†^; (Liang et al., 2024)M^(> 2 h/d) weekdays^; (Liang et al., 2024)M^(> 2 h/d) weekends^; (Shi et al., 2023)^(> 2h/d)†^; (Granger et al.,2017)^(≥ 4 h/d)^; (Nigg et al.,2015); (Spengler et al.,2014)M; (Spengler et al.,2014)F | O |  |  |  |  |  |  |  |  |  |  |  |  |
| Total recreational ST summary |  |  | 101 | 6 | 23 | 72 | 36 | 0 | 5 | 31 | 38 | 4 | 9 | 25 |
| **Study time** |  |  |  |  |  |  |  |  |  |  |  |  |  |  |
| Homework | (Yang et al., 2023)^(> 4 h/d) off shcool^ | - | 14 | 0 | 1 | 13 | 5 | 0 | 0 | 5 | 5 | 0 | 0 | 5 |
|  | (Martinez-Lopez et al.,2015)M^(≥ 4 h/d) weekdays^; (Martinez-Lopez et al.,2015)F^(≥ 4 h/d) weekdays^; (Martinez-Lopez et al.,2015)M^(≥ 4 h/d) weekend^; (Martinez-Lopez et al.,2015)F^(≥ 4 h/d) weekend^;(Herman et al.,2014)M^(> 1 h/d)^; (Herman et al.,2014)F^(> 1 h/d)^; (Liang et al., 2024)M^(> 2 h/d) weekdays^; (Liang et al., 2024)F^(> 2 h/d) weekdays^; (Liang et al., 2024)M^(> 2 h/d) weekends^; (Liang et al., 2024)F^(> 2 h/d) weekends^; (Yang et al., 2023)^(2–4 h/d) on shcool^; (Yang et al., 2023)^(> 4 h/d) on shcool^; (Yang et al., 2023)^(2–4 h/d) off shcool^ | O |  |  |  |  |  |  |  |  |  |  |  |  |
| Reading | (Herman et al.,2014)F^(> 1 h/d)^ | + | 2 | 1 | 0 | 1 | 1 | 0 | 0 | 1 | 1 | 1 | 0 | 0 |
|  | (Herman et al.,2014)M^(> 1 h/d)^ | O |  |  |  |  |  |  |  |  |  |  |  |  |
| Total study time summary |  |  | 16 | 1 | 1 | 14 | 6 | 0 | 0 | 6 | 6 | 1 | 0 | 5 |
| **Other SB** |  |  |  |  |  |  |  |  |  |  |  |  |  |  |
| Unspecified SB | (Husu et al.,2016); (Tebar et al.,2021)M^(≥ 4 h/d)^; (Tebar et al.,2021)F^(≥ 4 h/d)^; (Park, 2024)^study purpose in 2019^; (Park, 2024)^study purpose in 2020^; (Park, 2024)^study purpose in 2021^; (Park, 2024)^purpose other than study in 2019^; (Park, 2024)^purpose other than study in 2020^; (Park, 2024)^purpose other than study in 2021^ | - | 15 | 0 | 9 | 6 | 2 | 0 | 1 | 1 | 2 | 0 | 1 | 1 |
|  | (Smith et al.,2015); (Herman et al.,2014)M; (Herman et al.,2014)F; (Gonzalez-Alvarez et al., 2023)^little time^; (Gonzalez-Alvarez et al., 2023)^moderate time^; (Gonzalez-Alvarez et al., 2023)^lot of time^ | O |  |  |  |  |  |  |  |  |  |  |  |  |
| Telephone | (Li et al.,2018)M^(0–0.5 h/d)^; (Li et al.,2018)M^(0.5–1 h/d)^; (Li et al.,2018)M^(>1 h/d)^; (Li et al.,2018)F^(0–0.5 h/d)^; (Li et al.,2018)F^(0.5–1 h/d)^; (Li et al.,2018)F^(>1 h/d)^ | O | 6 | 0 | 0 | 6 | 3 | 0 | 0 | 3 | 3 | 0 | 0 | 3 |
| Computer (including homework) | (Moor et al.,2014)^(> 2 h/d)^ | - | 1 | 0 | 1 | 0 | 0 | 0 | 0 | 0 | 0 | 0 | 0 | 0 |
| Social media (including homework) | (Jodkowska et al.,2019)F^(> 2 h/d)^ | - | 1 | 0 | 1 | 0 | 0 | 0 | 0 | 0 | 1 | 0 | 1 | 0 |
| Total SB summary |  |  | 140 | 7 | 35 | 98 | 47 | 0 | 6 | 41 | 50 | 5 | 11 | 34 |

Notes. SB: Sedentary behavior; ST: screen time ; M: Male; F: Female; O: No significant association; +; Significant positive association; -: Significant negative association.

†: > 2 hours per day of recreational screen time weekly.

| Supplementary Table 3. Measurement details and findings from included studies (n=47). | | | | | | |
| --- | --- | --- | --- | --- | --- | --- |
| Articles | Mesurement contents | | |  | Findings | |
|  | PA variable use in the included articles (in review if change): Contents | SB variable use in the included articles (in review if change): Contents | SRH |  | PA and SRH | SB and SRH |
| Park, 2024 | PA was measured by two questions regarding VPA and muscle-strengthening exercises.  VPA: “Over the past seven days, how many days did you perform high-intensity PA (such as jogging, soccer, basketball, taekwondo, mountaineering, fast cycling, fast swimming, and carrying heavy objects) for 20 min or more?” on a six-point Likert scale (1 = None for the past 7 days to 6 = 5 times a week or over).  Muscle-strengthening exercise: “Over the past seven days, how many days did you carry out muscle-strengthening exercises such as push-ups, sit-ups, weightlifting, dumbbells, iron bars, and parallel bars to build muscle strength?” on a six-point Likert scale (1 = None for the past 7 days to 6 = 5 times a week or over | (1) SB for study purposes and (2) SB for purposes other than study: “Over the past seven days, how many hours per day on average did you spend sitting?” The answers were divided into two categories: ‘time spent sitting for study purposes’ and ‘sitting for purposes other than study.’ | “What do you think your health is usually like?” Responses were on a five-point Likert scale (1 = very healthy, 2 = healthy, 3 = normal, 4 = unhealthy, 5 = very unhealthy). |  | β, SE and *p* for better SRH.  PA: 2019:β = 0.262, SE=0.004, *p*＜0.05  2020:β = 0.257, SE=0.004, *p*＜0.05  2021:β = 0.272, SE=0.004, *p*＜0.05 | β, SE and *p* for better SRH.  SB for study purposes:  2019:β = -0.028, SE=0.001, *p*＜0.05  2020:β = -0.027, SE=0.001, *p*＜0.05  2021:β = -0.024, SE=0.001, *p*＜0.05  SB for purposes other than study:  2019:β = -0.051, SE=0.001, *p*＜0.05  2020:β = -0.041, SE=0.001, *p*＜0.05  2021:β = -0.044, SE=0.001, *p*＜0.05 |
| Moran et al., 2024 | MVPA: “On how many days in the last week did you do a total of at least an hour of MVPA?” (1 = Every day to 5 = Not at all). | NA | “How would you describe your health generally?” Responses were on a five-point Likert scale (1 = Excellent; 2 = Very good; 3 = Good; 4 = Fair; 5 = Poor). |  | β, SE and *p* for better SRH.  MVPA (≥ 1 h, 1–2 d/wk): β = 0.136, SE=0.041, *p*＜0.001  MVPA (≥1 h, 3–4 d/wk): β = 0.334, SE=0.04, *p*＜0.001  MVPA (≥1 h, 5–6 d/wk): β = 0.52, SE=0.042, *p*＜0.001  MVPA (≥1 h, 7 d/wk): β = 0.73, SE=0.042, *p*＜0.001 | NA |
| Liang et al., 2024 | (1) MVPA: how many days they engaged in at least 60 min of MVPA on weekdays and weekends, respectively. The answers were given on a 6-point scale (0 to 5 days) for weekdays, and on a 3-point scale (0 to 2 days) for weekends. (2) School-based PA: “How many hours a day did you spend in school PA? (including physical education, the class-break exercise or morning exercises, and extracurricular activities in school)”, on a 4-point scale (1 = never to 4 = more than an hour).  (3) Extracurricular PA (extracurricular VPA): “How many hours a day do you usually take physical exercise outside school class so that you get out of breath or sweat?” on a 4-point scale (1 = never to 4 = more than an hour). | (1) Screen time (recreational screen time): how many hours per day they watched television and used mobile phones/iPads and computers in their leisure time on weekdays and weekends, respectively.  (2) Homework: how many hours of homework they did each day in their spare time on weekdays and weekends, respectively.  The answers were given on a 5-point scale (1 = never to 5 = at least 3 hours). | ‘‘How would you describe your health?” options included: 1 = very good, 2 = good, 3 = fair, 4 = poor, and 5 = very poor. |  | OR and 95% CI for better SRH.  M: MVPA (≥1 h, 5d/wk vs. <5d/wk in weekdays): OR=1.70, 95% CI =1.35-2.13. MVPA (≥1 h, 2d/wk vs. <2d/wk in weekends): OR=2.00, 95% CI =1.59-2.50.  School-based PA (>1h/d vs. ≤1h/d): OR=1.49, 95% CI = 1.19-1.86. Extracurricular VPA (>1h/d vs. ≤1h/d): OR=1.82, 95% CI =1.42-2.34. F: MVPA (≥1 h, 5d/wk vs. <5d/wk in weekdays): OR=1.44, 95% CI =1.14-1.83. MVPA (≥1 h, 2d/wk vs. <2d/wk in weekends): OR=1.88, 95% CI =1.46-2.41. School-based PA (>1h/d vs. ≤1h/d): OR=1.08, 95% CI =0.86-1.34. Extracurricular VPA (>1h/d vs. ≤1h/d): OR=1.62, 95% CI =1.23-2.13. | OR and 95% CI for better SRH.  M: Recreational screen time (≤2h/d vs. >2h/d in weekdays): OR=0.98, 95% CI =0.78–1.24. Recreational screen time (≤2h/d vs. >2h/d in weekends): OR=1.05, 95% CI =0.86–1.29 Homework (≤2h/d vs. >2h/d in weekdays): OR=1.17, 95% CI =0.94–1.45 Homework (≤2h/d vs. >2h/d in weekends): OR=1.05, 95% CI =0.86–1.29 F: Recreational screen time (≤2h/d vs. >2h/d in weekdays): OR=1.38, 95% CI =1.10–1.74 Recreational screen time (≤2h/d vs. >2h/d in weekends): OR=1.40, 95% CI =1.14–1.71 Homework (≤2h/d vs. >2h/d in weekdays): OR=1.12, 95% CI =0.92–1.37 Homework (≤2h/d vs. >2h/d in weekends): OR=1.10, 95% CI =0.91–1.33 |
| Du et al., 2024 | NA | Internet use: “Do you use/Have you ever used the Internet?” If the answer was “yes,” Internet use was assigned a value of 1; otherwise, it was assigned a value of 0. | “How would you rate your physical health?” Responses were on a five-point Likert scale (1 = very healthy; 5 = unhealthy). |  | NA | z-values (robust standard errors) and *p* for poorer SRH.  Internet use: z-values(robust standard errors) = 0.067(2.186), *p*<0.05. |
| de Sales et al., 2024 | MVPA: to measure the weekly time in MVPA (min/wk of MVPA), we used a Brazilian validated list of 24 PA options, in which participants reported the daily duration and frequency over a usual week. | NA | “In general, how would you rate your health?” Students were given four options: very good, good, fair and poor. |  | β and 95% CI for better SRH.  MVPA (min/wk): β = 0.066, 95% CI= 0.039-0.092. | NA |
| Gonzalez-Alvarez et al., 2023 | PA: total PA was measured with the participant reports of the frequency they engaged in PA in their free time during the last week: I did not do PA in my free time, I sometimes (1–2 times last week), I often (3–4 times last week), I quite often (5–6 times last week), and I very often (7 or more times last week) did PA in my free time. | SB: adolescent SB was assessed through a question regarding typical sedentary habits at home (I spent almost none of my free time sitting, I spent little time sitting, I spent a moderate amount of time sitting, I spent a lot of time sitting, and I spent almost all of my free time sitting) | Adolescents classified their health as excellent, very good, good, fair, or poor by answering the question “In general, would you say your health is…” |  | OR and 95% CI for better SRH.  PA: Sometimes (1–2 times/wk vs. 0 time/wk), OR=1.04, 95% CI =0.64–1.68; Often (3–4 times vs. 0 time/wk), OR=1.29, 95% CI =0.79–2.13; Quite often (5–6 times vs. 0 time/wk), OR=2.02, 95% CI =1.22–3.36; Very often (7+ times vs. 0 time/wk), OR=2.17, 95% CI =1.20–3.92 | OR and 95% CI for better SRH.  SB: Little time vs. almost none, OR=1.09, 95% CI =0.59–2.02; Moderate time vs. almost none, OR=0.79, 95% CI =0.43–1.43; Lot of time vs. almost none, OR=0.69, 95% CI =0.36–1.30. |
| Wang et al., 2023 | MVPA: MVPA was assessed using the PA Scale developed by Sallis et al. and Andersen et al. The scale consisted of four items measured on a 5-point Likert scale, ranging from “strongly disagree (1)” to “strongly agree (5)”. The scale questions were structured as declarative sentences and employed a first-person inquiry method. For instance, the items included statements such as “During the past week, I actively participated in various forms of moderate PA, including tasks like sweeping and mopping, as well as engaging in sports such as volleyball, Ping-Pong, and similar activities”. | NA | The scale comprises five items, employing a 5-point Likert scale ranging from “poor (1)” to “very good (5)” to gauge the perceived status. For instance, a sample item reads as follows “How would you rate your mental health?” |  | β and *p* for better SRH.  MVPA: β = 0.347, *p* < 0.001 | NA |
| Shi et al., 2023 | MVPA: “During the past 7 days, how many days did you engage in MVPA for at least 60 min per day?” Adherence to the MVPA recommendation was indicated by reporting PA for 7 days. | Screen time (recreational screen time): “On both weekdays and weekends, how many hours did you typically spend doing the following activities in your leisure time, including watching TV or movies or DVDs, playing video games, and engaging in other screen-based activities?” Participants adhered to the screen time recommendation if they reported no more than 2 hours of screen time per day. | “In general, how do you consider your life satisfaction?” Response options were “very good”, “good”, “fair”, or “poor”. |  | OR and 95% CI for better SRH.  MVPA (≥1 h/d vs. <1 h/d): OR=4.96, 95% CI=2.82-8.72 | OR and 95% CI for better SRH.  Recreational screen time (≤2h/d vs. >2h/d): OR=1.06, 95% CI=0.88-1.28 |
| Yang et al., 2023 | (1) Exercise: the average daily physical exercise time was calculated by replacing the value of each case that answers “more than 4 hours” with 5 hours. After that, it was operationalized into three categories: 1. less than or equal to quarter an hour, 2. more than quarter an hour less than or equal to half an hour, and 3. more than half an hour. (2) Housework: for housework time, this study replaced the case values over 4 hours with 5 hours and over 60 min with 60 min, thus calculating the average daily housework time, and then transformed it into three categories, “1. less than or equal to half an hour, 2. more than half an hour less than or equal to 1 hour, 3. more than 1 hour”. | (1) TV: the questionnaire asked respondents how much time they watch TV per day, and the corresponding options were “1. None to 6. more than 4 hours”. Next, the respondents were asked how much time they spent watching TV on weekends, and the corresponding options were “1= none to 6= more than 8 hours”.  (2) Online and playing games: students were asked about the amount of time they spent online and playing games each day, and the corresponding options were set the same as those for time spent watching TV each day. (3) School study time (homework time on school) and (4) off campus study and homework time (homework time off school): the homework time on campus and off campus per day was measured in the same way as the screen time, divided into “1. less than or equal to 2 to 3 hours. more than 4 hours”. | “How is your overall health now”, and the corresponding options were “1. very bad, 2. not good, 3. fair, 4. better, 5. very good”. |  | OR and *p* for better SRH.  Exercise:  0.25–0.5 h/d vs. ≤0.25 h/d, OR = 0.146, *p* < 0.05; >0.5 h/d vs. ≤0.25 h/d, OR=0.612, *p* < 0.05. Housework: 0.5–1 h/d vs. ≤0.5 h/d, OR= 0.089, *p* < 0.1; >1 h/d vs. ≤0.5 h/d, OR=0.146, *p* < 0.05. | β and *p* for better SRH.  TV watching:  1–2 h/d vs. ≤1 h/d, β =0.098, *p* < 0.05; >2 h/d vs. ≤1 h/d, β =0.168, *p* < 0.05. Online and game: 1–2 h/d vs. ≤1 h/d, β =−0.117, *p* < 0.05; >2 h/d vs. ≤1 h/d, β =−0.206, *p* < 0.05. Homework time on school: 2–4 h/d vs. ≤2 h/d, β =0.104, *p* > 0.1; >4 h/d vs. ≤2 h/d, β =0.008, *p* > 0.1. Homework time off school: 2–4 h/d vs. ≤2 h/d, β =−0.113, *p* > 0.1; >4 h/d vs. ≤2 h/d, β =−0.223, *p* < 0.05. |
| Kyan et al., 2022 | MVPA: PA was measured using Patient-Centered Assessment and Counselling for Exercise plus Nutrition. This was developed to identify the extent to which young people achieved the current guidelines, which is a minimum of 60 min of MVPA per day of the week. | Screen time (recreational screen time): “How many hours a day do you usually spend watching TV at home on weekdays?”; “How many hours a day do you usually use your personal computer (including smartphone or tablet), excluding the time when these devices were used for learning, to play computer games (such as TV game, computer game, and mobile game) at home on weekdays?” Possible answers to each question were “never,” to “more than 5 h/d.” | SRH was assessed by answering five possible answers (“Strongly disagree,” “disagree,” “neither agree nor disagree,” “agree,” “Strongly agree”) to “I'm healthy at present.” |  | OR and 95% CI for better SRH.  MVPA (≥1 h/d vs. <1 h/d): elementary school students, OR =1.86, 95% CI= 0.85–4.03; Junior high school students, OR =2.79, 95% CI= 1.22–6.36. | OR and 95% CI for better SRH.  Recreational screen time (≤ 2 h/d vs. >2h/d): elementary school students, OR =1.19, 95% CI= 0.68–2.08; Junior high school students, OR =1.52, 95% CI= 0.80–2.91. |
| Karchynskaya et al., 2022 | (1) MVPA: asking adolescents about the number of days over the past week that they were physically active for a total of at least an average of 60 min per day. The question was preceded by an explanatory text that defined moderate-to-vigorous activity as ‘any activity that increases your heart rate and makes you get out of breath some of the time, offering examples of such activities (running, inline skating, cycling, dancing, swimming, ice skating etc). Responses could vary from 0 to 7 days per week.  (2) Organized leisure time activities (Organized leisure time sport activities): “Are you doing any of these organized activities in your free time?” with response categories‘yes and‘no’. In this research, we used only two categories: (i) organized team sport activities (e.g. football, basketball and volleyball); and (ii) organized individual sport activities (e.g. tennis, gymnastics and karate). | NA | “Would you say your health is……?” with four possible answers: ‘poor’, ‘fair’, ‘good’/‘excellent’. |  | OR and 95% CI for better SRH.  MVPA: ≥1 h/d, 7 d/wk vs. < 7 d/wk, OR=2.7, 95% CI= 1.9–3.8; ≥1 h/d, 5–7 d/wk vs. <5 d/wk, OR=2.6, 95% CI= 1.9–3.6. Organized leisure time sport activities (Yes vs. no): OR=2.7, 95% CI= 1.9–3.9. | NA |
| Wang et al., 2022 | VPA: “how often did you do a workout by which you sweat or get out of breath after class?” The answers included never, less than once a month, once a month, once a week, 2 to 3 times a week, 4 to 6 times a week and every day. | NA | “Would you say your health is…?”—and a four-point response scale was used including the categories excellent, good, fair and poor. |  | OR and 95% CI for poorer SRH.  VPA: 4–6 times/wk vs. everyday, OR=1.30, 95% CI= 1.25-1.35; 2–3 times/wk vs. everyday, OR=1.69, 95% CI= 1.63-1.76; 1 times/wk vs. everyday, OR=1.95, 95% CI= 1.86-2.04; Once a month vs. everyday, OR=2.14, 95% CI= 2.00-2.29; Less than once a month vs. everyday, OR =2.29, 95% CI= 2.15-2.45; Never vs. everyday, OR =2.08, 95% CI= 1.94-2.24. | NA |
| de Fátima Guimarães et al., 2022 | MVPA: girls self-reported their MVPA levels. Total min per week were used to calculate a proxy of their daily amount of MVPA. These variables were analyzed based on the Canadian 24-Hour Movement Guidelines for children and youth: at least 60 min of MVPA. The online questionnaire was available in both French and English (questions and scales used for the present analyses are presented as [supplementary material](https://journals.sagepub.com/doi/full/10.1177/1559827620964764?casa_token=sIrJv1rYxcwAAAAA%3AvxiMStA_02aUGiXfOjtJtvc4TGxCKafqGayzC4K_pOB2Up3_JSLtpyVW7CbAXVWIu7OiFfbSRp1i#supplementary-materials), available online). | Screen time (recreational screen time): screen time was the total hours and min per week self-reported. These variables were analyzed based on the Canadian 24-Hour Movement Guidelines for children and youth: no more than 2 hours of recreational screen time. The online questionnaire was available in both French and English (questions and scales used for the present analyses are presented as [supplementary material](https://journals.sagepub.com/doi/full/10.1177/1559827620964764?casa_token=sIrJv1rYxcwAAAAA%3AvxiMStA_02aUGiXfOjtJtvc4TGxCKafqGayzC4K_pOB2Up3_JSLtpyVW7CbAXVWIu7OiFfbSRp1i#supplementary-materials), available online). | Participants had 5 options to describe their perceived health: excellent, very good, good, fair, and poor. |  | χ2 and *p* for SRH.  MVPA: ≥ 1 h/d, 16.1% for excellent/very good SRH, 7.4% for good/fair/poor SRH. < 1 h/d, 83.9% for excellent/very good SRH, 92.6 % for good/fair/poor SRH. χ2 = 4.412, *p* = 0.03. | χ2 and *p* for SRH.  Recreational screen time: ≤ 2 h/d=41.3% for excellent/very good SRH, 27.8% for good/fair/poor SRH;  > 2 h/d =58.7% for excellent/very good SRH, 72.2% for good/fair/poor SRH. χ2 = 5.060, *p* = 0.02. |
| Pierannunzio et al., 2022 | (1) MVPA: “Over the past 7 days, on how many days were you physically active for a total of at least 60 min per day?” (the so-called “MVPA” with possible answers: 0 = 0 days to 7 = seven days).  (2) VPA (out of school VPA): “Outside school hours: how often do you usually exercise in your free time so much that you get out of breath or sweat?” (the so-called “VPA” with possible answers: 0 = never to 6 = every day). | NA | “Would you say your health is…?”. The four possible answers were dichotomized into “excellent/good” vs. “fair/poor”. |  | OR and 95% CI for better SRH.  M: 11 Years Old: MPVA (≥1 h/d, ≥ 4d/wk vs. <4d/wk), OR = 2.23, 95% CI=1.62–3.07. VPA (≥ 2d/wk vs. <2d/wk), OR =1.31, 95% CI= 0.98–1.74. 13 Years Old: MPVA (≥1 h/d, ≥ 4d/wk vs. <4d/wk), OR = 1.56, 95% CI=1.20–2.03. VPA (≥ 2d/wk vs. <2d/wk), OR =1.75, 95% CI= 1.30–2.34. 15 Years Old: MPVA (≥1 h/d, ≥ 4d/wk vs. <4d/wk), OR = 1.57, 95% CI=1.21–2.05. VPA (≥ 2d/wk vs. <2d/wk), OR =2.48, 95% CI= 1.92–3.21. F: 11 Years Old: MPVA (≥1 h/d, ≥ 4d/wk vs. <4d/wk), OR = 1.20, 95% CI=0.89–1.62. VPA (≥ 2d/wk vs. <2d/wk), OR =1.48, 95% CI= 1.11–1.97. 13 Years Old: MPVA (≥1 h/d, ≥ 4d/wk vs. <4d/wk), OR = 1.53 95%, CI=1.14–2.06. VPA (≥ 2d/wk vs. <2d/wk), OR =1.48, 95% CI= 1.15–1.91. 15 Years Old: MPVA (≥1 h/d, ≥ 4d/wk vs. <4d/wk), OR = 1.37, 95% CI=1.09–1.71. VPA (≥ 2d/wk vs. <2d/wk), OR =1.67, 95% CI= 1.37–2.03. | NA |
| Badura et al., 2021 | Organized leisure time activities (Organized leisure time sports activities): adolescents’ participation (yes/no) in six particular types of activities was measured: team sports, individual sports, arts, youth organisations, afterschool clubs/leisure centres and religious activities. Patterns of participation were measured by distinguishing four categories: (1) no participation in any of activities, (2) participation in non-sport activities only (ie, any activities except for team and/or individual sports), (3) participation in sport activities only (ie, only team and/or individual sports) and (4) participation in both non-sport and sport activities. Note: in the review, we only extract (3) sport activities results. | NA | “Would you say your health is…?” with four response options (poor, fair, good and excellent) |  | OR and 95% CI for better SRH.  organised leisure-time sport activities (yes vs. no), OR=1.67, 95% CI=1.55-1.79. | NA |
| Tebar et al., 2021 | PA: total PA was computed as weekly min of PA scores across the different domains (active transport, leisure PA and physical education classes), which was categorized in 1: 0–299 min/week and 2: ≥300 min/week. | SB: the participants were asked about the total time per day expended in sitting position activities, which was categorized in “low” (less than 4 hours) and “high” (4 hours and more). | “In general, how do you consider your health?” Responses were on a 5-point Likert-type scale (1 = very good; 2 = good; 3 = Regular; 4 = bad; 5 = very bad) |  | OR and 95% CI for better SRH.  M: PA (≥300 min/wk vs. <300 min/wk), OR=1.12, 95% CI= 1.04–1.21. F: PA (≥300 min/wk vs. <300 min/wk), OR=1.01, 95% CI= 0.93-1.08. | OR and 95% CI for better SRH.  M: SB (<4 h/d vs.≥4 h/d), OR= 1.42, 95% CI= 1.32–1.54. F: SB (<4 h/d vs.≥4 h/d), OR= 1.32, 95% CI= 1.23–1.41. |
| Moral-García et al., 2020 | PA: to assess the level of PA practice, the International PA Questionnaire (IPAQ) was used, in its adapted version for European adolescents IPAQ-A (including Spanish teenagers), which obtained appropriate validity. For this study, the PA field was chosen during leisure, sports and leisure time (divided into walking PA, moderate PA and VPA), which allowed the subjects to be classified as active and sedentary. | NA | “Your health is…” Responses were on a 4-point Likert-type scale (poor, reasonable, good or excellent) |  | ß and *p* for better SRH. PA: ß = 0.142, *p* = 0.014. | NA |
| Marques et al., 2019 | PA: they were asked to rate the number of days over the past week that they were physically active, for a total of at least 60 min per day. Answers were given on an 8-point scale (0 = none to 7 = daily). | Screen time (recreational screen time): watching television, playing videogames and using computers were selected as the screen-based SB. Adolescents were asked to indicate the average time (hours per day) they spent on each activity. Total screen-based behaviour was calculated by the sum of these three SB. The total sum of screen-based SB was dichotomised into ≥2 h and <2 h daily. | “You would say your health is…?” Responses were on a 4-point scale (poor, fair, good and excellent). |  | Mean, 95% CI and *p* for better SRH.  PA (≥ 1 h, ≤6 d/wk), Mean=3.2, 95% CI= 3.2-3.2; PA (≥ 1 h, 7 d/wk), Mean= 3.5, 95% CI=3.5-3.6, *p*<0.001. | Mean, 95% CI and *p* for better SRH.  Recreational screen time (≥2 h/d), Mean= 3.2, 95% CI= 3.2-3.3; Recreational screen time (<2 h/d), Mean=3.2, 95% CI=3.2-3.3, *p*=0.426. |
| Jodkowska et al., 2019 | (1) MVPA: “Over the past 7 days, on how many days were you physically active for a total of at least 60 min per day?”. MVPA was dichotomized into two categories: recommended PA level (7 days a week) and PA below recommendations.  (2) VPA (out of school VPA): “Outside school hours: how often do you usually exercise in your free time so much that you get out of breath or sweat?” –the indicator was dichotomous: recommended VPA level 2-3 times a week or more, and below the recommendations (VPA< 2-3 times a week)  (3) Physical education: “In how many physical education classes did you participate in the previous school year?” Participation in physical education classes was analysed in a dichotomous division: regularly (all and almost all classes), and irregularly (less often than in previous category). | (1) TV/DVD (recreational screen time): “How many hours a day, do you usually spend in your free time: watching TV, videos (including YouTube or similar services), DVDs, and other entertainment on a screen? (2) Social media: “How many hours a day do you usually spend in your free time using electronic devices, such as computers, tablets (like iPad) or smartphones for other purposes, for example, homework, emailing tweeting Facebook, chatting surfing the internet? In subsequent analyses, a dichotomous split was applied: less than two hours and over two hours. | Would you say that your health is: excellent, good, fair, poor. |  | OR and 95% CI for better SRH.  Physical education (attend all and almost all classes vs. less often than in previous category), OR=1.82, 95% CI=1.40-2.38. OVPA (≥ 2 d/wk vs. < 2 d/wk), OR=1.50, 95% CI=1.23-2.03. MVPA (≥ 1 h/d, 7d/wk vs. < 7 d/wk), OR=1.66, 95% CI=1.12-2.45. | OR and 95% CI for better SRH.  Recreational screen time (≤2 h/d vs. >2 h/d, OR=1.38, 95% CI=1.08-1.79. Social media (≤2 h/day vs. >2 h/d), OR=1.50, 95% CI=1.17-1.93. |
| Werneck et al., 2018 | NA | (1) TV, and (2) computer/video game: screen time were assessed through four questions asking about how many hours a day were spent watching television and how many hours a day were spent using computer and video games on weekdays and weekends. | “In general, how do you consider your health?” Responses were on a 4-point Likert-type scale ranging from 1 = bad to 4 = excellent. |  | NA | r and *p* for better SRH.  M: Computer and video game (h/d): r = −0.14, *p* < 0.05. TV watching (hours per day): r =−0.06, *p* >0.05) F: Computer and video game (h/d): r =−0.02, *p* >0.05. TV watching (hours per day): r =−0.03, *p* >0.05. |
| Hwang et al., 2018 | (1) Moderate PA: performed PA that caused the heart rate to increase or the subject to be short-winded for at least 60 min a day during the past 7 days.  (2) VPA: performed strenuous PA that caused the subject to be completely short-winded or the body to sweat for at least 20 min a day during the past 7 days.  (3) Muscle-strengthening: performed muscle-strengthening exercise during the past 7 days. | NA | “How would you rate  your health status?” using one of the following responses: “Very healthy”, “healthy”, “so-so”,  “unhealthy”, and “very unhealthy.” |  | OR and 95% CI for poorer SRH.  VPA (≥3 d/wk vs. <3 d/wk), OR=0.59, 95% CI=0.55-0.65. Muscle-strengthening exercise (≥3 d/wk vs. <3 d/wk), OR=0.57, 95% CI=0.52-0.63. Moderate PA (≥5 d/wk vs. <5 d/wk, OR=1.01, 95% CI=0.89-1.15. | NA |
| Li et al., 2018 | PA: PA included four response categories: Frequency of PA: nearly every day, ≥3 times per week, 1–2 times per week, <1 time per week. | (1) TV, (2) video game, (3) telephone usage, (4) mobile phone e-mail usage and (5) computer usage: questions on sedentary lifestyles included, television-viewing time (≤2 h, 2–3 h, 3–4 h, >4 h for 24 h), video game playing time (0, 0–1 h, 1–2 h, 2–3 h, >3 h for 24 h), telephone usage (0, 0–0.5 h, 0.5–1 h, >1 h for 24 h), mobile phone e-mail usage (≤2 h, 2–3 h, 3–4 h, >4 h for 24 h), and personal computer usage (≤2 h, 2–3 h, 3–4 h, >4 h for 24 h). | “In the past 4 weeks, how did you assess your health?” The answer included five options: excellent, very good, good, fair, and poor. |  | OR and 95% CI for poorer SRH.  M: PA (≤2 times/wk vs. ≥3 times/wk), OR=1.14, 95% CI=0.89-1.45. F: PA (≤2 times/wk vs. ≥3 times/wk), OR=1.16, 95% CI=0.97-1.38. | OR and 95% CI for poorer SRH.  M: TV: 2–3 h/d vs. ≤2 h/d, OR=0.9, 95% CI=0.71-1.13; 3-4 h/d vs. ≤2 h/d, OR=0.89, 95% CI=0.65-1.23; >4 h/d vs. ≤2 h/d, OR=1.06, 95% CI=0.72-1.56. Video game: 0-1 h/d vs. 0h/d, OR=0.82, 95% CI=0.66-1.03; 1–2 h/d vs. 0h/d, OR=0.92, 95% CI=0.71-1.21; 2–3 h/d vs. 0h/d, OR=0.84, 95% CI=0.53-1.32; >3h/d vs. 0h/d, OR=0.78, 95% CI=0.42-1.46. Telephone using: 0–0.5 h/d vs. 0h/d, OR=0.93, 95% CI=0.75-1.14; 0.5–1 h/d vs. 0h/d, OR=1.17, 95% CI=0.7-1.96; >1 h/d vs. 0h/d, OR= 0.59, 95% CI=0.28-1.21. Mobile phone e-mail using: 2-3 h/d vs. ≤2 h/d, OR=0.92, 95% CI=0.64-1.32; 3-4 h/d vs. ≤2 h/d, OR=0.77, 95% CI=0.42-1.41; >4 h/d vs. ≤2 h/d, OR=1.27, 95% CI=0.80-2.04.  Computer: 2-3 h/d vs. ≤2 h/d, OR=1.28, 95% CI=0.86-1.92; 3-4 h/d vs. ≤2 h/d, OR=1.34, 95% CI=0.72-2.5; >4 h/d vs. ≤2 h/d, OR=1.91, 95% CI=0.99-3.68.  F: TV: 2–3 h/d vs. ≤2 h/d, OR=0.92, 95% CI=0.74-1.13; 3-4 h/d vs. ≤2 h/d, OR=0.71, 95% CI=0.52-0.96; >4 h/d vs. ≤2 h/d, OR=1.12, 95% CI=0.82-1.54. Video game: 0-1 h/d vs. 0h/d, OR=1.37, 95% CI=1.05-1.79; 1–2 h/d vs. 0h/d, OR=1.17, 95% CI=0.71-1.93; 2–3 h/d vs. 0h/d, OR=1.03, 95% CI=0.45-2.33; >3h/d vs. 0h/d, OR=2.36, 95% CI=0.85-6.54. Telephone using: 0–0.5 h/d vs. 0h/d, OR=1.01, 95% CI=0.84-1.23; 0.5–1 h/d vs. 0h/d, OR=1.27, 95% CI=0.85-1.9; >1 h/d vs. 0h/d, OR=1.48, 95% CI=0.92-2.4. Mobile phone e-mail using: 2-3 h/d vs. ≤2 h/d, OR=1.17, 95% CI=0.9-1.5; 3-4 h/d vs. ≤2 h/d, OR=1.01, 95% CI=0.7-1.47; >4 h/d vs. ≤2 h/d, OR=1.53, 95% CI=1.14-2.06. Computer: 2-3 h/d vs. ≤2 h/d, OR=1.15, 95% CI=0.8-1.67; 3-4 h/d vs. ≤2 h/d, OR=1.53, 95% CI=0.86-2.71; >4 h/d vs. ≤2 h/d, OR=2.83, 95% CI=1.39-5.76. |
| Curtin et al., 2018 | (1) School-based organized sports: “How often do you participate in school-based organized sports?”.  (2) After- school organized sports or Saturday school sports: “How often do you participate in after- school organized sports or Saturday school sports?”.  (3) Sport outside of school: “How often do you participate in outside of school sports?”.  Responses were dichotomized into those who participated versus those who did not participate in each type of PA at least once a week. | NA | “How do you perceive your current health status?” rated on a 5-point Likert scale from 1 (very unhealthy) to 5 (very healthy). |  | OR and 95% CI for better SRH.  School-based organized sports (≥ 1 times/wk vs. 0times/wk), OR=0.83, 95% CI=0.29-2.40. After-school organized sports or Saturday school sports (≥ 1 times/wk vs. 0times/wk), OR=0.66, 95% CI=0.15-2.96. Outside of school sports (≥ 1 times/wk vs. 0times/wk), OR=0.98, 95% CI=0.44-2.18. | NA |
| Silva et al., 2017 | NA | (1) TV and (2) video games/computer ues: adolescents’ screen time was accessed through a self-reported questionnaire, which contained four questions asking how many hours a day the adolescent spent watching television and how many hours a day he/she spent using a computer and videogames, on weekdays and weekends. For analyses, screen time was separated into two categories: TV-viewing (0 to 2.0 h/d, 2.1 to 4.0 h/d and > 4h/d) and video games/computer use (0 h/d, 0.1 to 2.0 h/d, 2.1 to 4.0 h/d and > 4h/d) | “In general, how do you consider your health?”, with four possibilities ranging from “poor” to “excellent”. |  | NA | OR and 95% CI for poorer SRH.  M: TV in weekdays: 2.1 to 4.0 h/d vs. 0 to 2.0 h/d, OR=1.05, 95% CI=0.57-1.92; > 4.0 h/d vs. 0 to 2.0 h/d, OR=1.46, 95% CI=0.84-2.53. TV in weekends: 2.1 to 4.0 h/d vs. 0 to 2.0 h/d, OR=2.07, 95% CI=1.12-3.84; > 4.0 h/d vs. 0 to 2.0 h/d, OR=1.86, 95% CI=1.05-3.31. Video games/computer in weekdays: 0.1 to 2.0 h/d vs. 0 h/d, OR=0.98, 95% CI=0.44-2.2; 2.1 to 4 h/d vs. 0 h/d, OR=0.87, 95% CI=0.38-1.97; > 4.0 h/d vs. 0 h/d, OR=1.62, 95% CI=0.75-3.48. Video games/computer in weekends: 0.1 to 2.0 h/d vs. 0 h/d, OR=0.99, 95% CI=0.49-2.03; 2.1 to 4 h/d vs. 0 h/d, OR=1.07, 95% CI=0.52-2.21; > 4.0 h/d vs. 0 h/d, OR=0.77, 95% CI=0.39-1.52. F: TV in weekdays: 2.1 to 4.0 h/d vs. 0 to 2.0 h/d, OR=0.84, 95% CI=0.52-1.38; > 4.0 h/d vs. 0 to 2.0 h/d, OR=1.08, 95% CI=0.7-1.67. TV in weekends: 2.1 to 4.0 h/d vs. 0 to 2.0 h/d, OR=1.12, 95% CI=0.69-1.81; > 4.0 h/d vs. 0 to 2.0 h/d, OR=1.44, 95% CI=0.94-2.2. Video games/computer in weekdays: 0.1 to 2.0 h/d vs. 0 h/d, OR=0.48, 95% CI=0.3-0.79; 2.1 to 4 h/d vs. 0 h/d, OR=0.57, 95% CI=0.35-0.95; > 4.0 h/d vs. 0 h/d, OR=0.61, 95% CI=0.37-0.99. Video games/computer in weekends: 0.1 to 2.0 h/d vs. 0 h/d, OR=0.5, 95% CI=0.3-0.82; 2.1 to 4 h/d vs. 0 h/d, OR=0.72, 95% CI=0.44-1.19; > 4.0 h/d vs. 0 h/d, OR=0.76, 95% CI=0.48-1.22. |
| Lachytova et al., 2017 | PA (VPA): “How often do you do sports (such that you breathe hard and sweat) in your free time?” Answering options were: every day (1), four to six times a week (2), two three times a week (3), once a week (4), once a month (5), less than once a month (6) and never (7). | (1) TV: “How much time do you spend watching television during an average school day?”  (2) Computer: “How much time do you spend on a computer during an average school day?”  The answers were dichotomized into two or more hours watching TV and using a computer (1) and less than 2 hours watching TV and using a computer (2) | “Would you say your health is…?” Answering options were: excellent (1), very good (2), good (3), not bad (4) and bad (5). |  | OR and 95% CI for better SRH.  VPA: 1 time/wk vs. 0 time/wk, OR=0.94, 95% CI=0.35-2.55; 2-3 time/wk vs. 0 time/wk, OR=1.35, 95% CI=0.57-3.18; 4-6 time/wk vs. 0 time/wk, OR=3.67, 95% CI=1.14-11.78; everday vs. 0 time/wk, OR=8.04, 95% CI=1.62-39.85. | OR and 95% CI for better SRH.  TV ＜2h/d vs. ≥2h/d, OR=2.56, 95% CI=1.37-4.79.  Computer ＜2h/day vs. ≥2h/day, OR=0.85, 95% CI=0.39-1.81. |
| Granger et al., 2017 | MVPA: “Over the past 7 days, on how many days were you physically active for a total of at least 60 min per day?” Participants who achieved this duration on all 7 days were assigned to the ‘active group’. PA on 6 days or fewer were assigned to the ‘inactive group’ | SB (recreational screen time): SB was assessed by hours a day watching TV or using a computer or a video game, and analyzed with two levels: ≥4 h/day, <4 h/day. | A standard validated 5-point Likert rating scale (excellent, very good, good, fair or poor) was used to assess health status. |  | OR and 95% CI for better SRH.  MVPA (≥ 1 h/d, 7d/wk vs. ≤6d/wk), OR=1.607, 95% CI=1.245-2.074. | OR and 95% CI for better SRH.  Recreational screen time (<4 h/d vs. ≥4 h/d, OR=1.101, 95% CI=0.952-1.274. |
| Husu et al., 2016 | PA was measured with Hookie AM 20-accelerometer (Traxmeet Ltd, Espoo, Finland) for seven days.  (1) Steps: steps were identified from the vertical impacts of the foot strikes. For step detection was used a fixed threshold, which requires about 3 kilometers per hour walking speed to detect every step.  (2) Light PA: light PA was defined as activity corresponding 1.5 − 2.9 MET.  (3) MVPA: moderate activity as 3.0–5.9 MET and vigorous activity 6 MET and over. | SB was measured with Hookie AM 20-accelerometer (Traxmeet Ltd, Espoo, Finland) for seven day.  SB: according to definition of SB, time spent in sitting and reclining positions were combined to indicate SB, while standing still position was analysed separately. | Perceived health status was assessed by asking children to report whether they perceived their health status as excellent, good, fair or poor. |  | OR and 95% CI for better SRH.  Steps per day, OR=1.08, 95% CI= 1.02-1.15. Light PA (h/d), OR=1.69, 95% CI=1.08-2.65. MVPA (min/d), OR=1.05, 95% CI=1.00-1.11. | OR and 95% CI for better SRH.  SB (h/d), OR=0.77, 95% CI=0.66-0.89. |
| Smith et al., 2015 | PA (out of school PA): this quesionnaire assesses the accumulated time spent physically active or sedentary respectively over the previous seven days outside of school. | SB: the total time involved in sedentary activities, including screen time, was also estimated for outside of school. | Participants were asked to rate their own health in general  responses were dichotomised to fair/poor/very poor as opposed to good/very good. |  | β and 95% CI for poorer SRH.  out-of-school PA, β= 0.25, 95% CI=-1.09-1.58. | β and 95% CI for poorer SRH.  SB, β=1.85, 95% CI=−0.18-3.88. |
| Meireles et al., 2015 | PA: PA was based on the instrument of the National Health Survey of School. We calculated the time of PA accumulated in the last seven days using a combination of the following activities: commuting to school on foot or by bicycle, physical education classes at school and other extracurricular PA. PA over the last seven days (active: 300 min or more or inactive/insufficiently active: up to 299 min). | (1) TV: time spent watching TV (less than 1 h/d, 2 h/d; or 3 h/d or more).  (2) Videogames or uses the computer: time spent per day playing videogames or on the computer (less than 1 hour, 2 hours; or 3 hours or more). | “In general, do you consider your health: very good, good, reasonable, poor or very poor?” |  | OR and 95% CI for poorer SRH.  Active PA (≥ 300 min/wk) vs. insufficiently PA (unspecified duration), OR=1.43, 95% CI=0.89-2.3; Active PA (≥ 300 min/wk) vs. inactive (unspecified duration), OR=2.31, 95% CI=1.15-4.69. | *p* value obtained by chi-square test for SRH.  TV: ≤1h/d, poor SRH (%)= 21.0, good SRH (%)=27.0; 2 h/d, poor SRH (%)=16.8, good SRH (%)=16.4; ≥ 3 h/d, poor SRH (%)=62.2, good SRH (%)=56.6. *p* =0.365. Video games/computer: ≤1h/d, poor SRH (%)=53.8, good SRH (%)=52.7; 2 h/d, poor SRH (%)=10.9, good SRH (%)=15.8; ≥ 3 h/d, poor SRH (%)=35.3, good SRH (%)=32.1. *p* =0.438. |
| Martinez-Lopez et al., 2015 | MVPA: two items were used to ask about PA levels (from moderate- to-vigorous): at least one hour a day in the previous week and a typical week. The response scale was the same for both items: 1=no days to 8=seven days. A mean of the responses to both items was used to dichotomize inactive or low PA level (1—5) and active or high PA level (6—8) participants. | (1) TV, (2) computer and (3) homework: participants responded to six items indicating the number of hours a day that they watch TV on weekdays, TV at weekends, computer on weekdays, computer on weekends, homework on weekdays, and homework on weekends. The response scale presented 9 options, from 1 = 0 hours to 9 = seven hour. The responses were classified as <2hours, 2—3hours, and ≥4hours per day. | “You would say that your health is…”, and responses included: 1 = poor, 2 = reasonable, 3 = good, and 4 = excellent. |  | OR and 95% CI for poorer SRH.  M: MVPA (≥ 1 h/d, ≤ 4 d/wk vs. > 4 d/wk, OR=1.436, 95% CI=1.102-1.871. F:  MVPA (≥ 1 h/d, ≤ 4 d/wk vs. > 4 d/wk, OR=1.467, 95% CI=1.023-2.103. | OR and 95% CI for poorer SRH.  M: TV (≥ 4 h/d vs. ＜4 h/d): in weekdays, OR=1.117, 95% CI=0.98-1.413; In weekend, OR=1.039, 95% CI=0.881-1.225.  Computer (≥ 4 h/d vs. ＜4 h/d): in weekdays, OR=1.217, 95% CI=1.022-1.45; In weekend, OR=0.932, 95% CI=0.796-1.092 Homework (≥ 4 h/d vs. ＜4 h/d):in weekdays, OR=11.012, 95% CI=0.834-1.227; In weekend, OR=0.942, 95% CI=0.775-1.145. F:  TV (≥ 4 h/d vs. ＜4 h/d): in weekdays, OR=0.921, 95% CI=0.741-1.144; In weekend, OR=0.889, 95% CI=0.727-1.087.  Computer (≥ 4 h/d vs. ＜4 h/d):in weekdays, OR=1.014, 95% CI=0.825-1.248; In weekend, OR=0.941, 95% CI=0.777-1.139. Homework (≥ 4 h/d vs. ＜4 h/d):in weekdays, OR=1.025, 95% CI=0.819-1.284; In weekend, OR=1.079, 95% CI=0.861-1.353. |
| Kantomaa et al., 2015 | PA (Out of school PA): the amount of PA outside school hours was evaluated separately for MVPA and light PA at age 16 years by asking participants, “How many hours a week all together do you participate in (a) brisk and (b) light PA outside school hours?” In the questionnaire, the term “brisk” was defined as MVPA. In addition, the adolescents were asked about their daily time spent in physically active commutes to and from school. The response alternatives (not at all to at least 1 hour per day) were multiplied by 5 (5 school days a week) to correspond to 0, 1, 2.5, 3.75, and 5 hours per week. An MET intensity value of 3 METs was used for light PA, 5 METs for brisk PA, and 4 METs for all types of commuting PA in the calculations. MET hours were divided into sex-specific thirds: (1) high (the highest tertile), (2) average (the middle tertile), and (3) low (the lowest tertile). | NA | “How would you  describe your health at the moment?” The response alternatives  were: (1) very poor, (2) poor, (3) fair, (4) good, and (5) very good. |  | OR and 95% CI for better SRH.  M:  Out-of-school PA: the middle tertile vs. the lowest tertile, OR=1.33, 95% CI=0.81-2.18; the highest tertile vs. the lowest tertile, OR=5.50, 95% CI=3.16-9.58 F:  Out-of-school PA: the middle tertile vs. the lowest tertile, OR=1.7, 95% CI=0.95-3.02; the highest tertile vs. the lowest tertile, OR=4.25, 95% CI=2.37-7.61 | NA |
| Herman et al., 2015 | PA: PA was assessed using an adaptation of the Minnesota Leisure Time PA Questionnaire. Respondents were asked about their participation in 21 specified activities, plus up to 3 additional volunteered activities, indicating participation frequency in the past 3 months and average session duration. Average daily energy expended during leisure time PA was calculated, weighting activities by their MET values. A PA index categorized respondents as active (≥3.0 kilocalories per kilogram per day), moderately active (1.5–2.9 kilocalories per kilogram per day) or inactive (<1.5 kilocalories per kilogram per day). | Screen time (recreational screen time): “In a typical week in the past 3 months, how much time did you usually spend: ... on a computer, including playing computer games and using the Internet?” (not including time spent at work or at school)… playing video games, such as XBOX, Nintendo and Playstation? ... watching television or videos?” Responses were summed to give a pre-categorized total weekly screen time, from which an upper cut-off of 14 h/wk was used to denote the cut-point of 2 h/d. | “Would you say your health in general is excellent, very good, good, fair, or poor?” |  | OR and 95% CI for poorer SRH.  M: PA: 1.5–2.9 kilocalories per kilogram per day vs. ≥ 3.0 kilocalories per kilogram per day, OR=1.59, 95% CI=1.33-1.9; <1.5 kilocalories per kilogram per day vs. ≥ 3.0 kilocalories per kilogram per day, OR=2.09, 95% CI=1.75-2.50. F: PA: 1.5–2.9 kilocalories per kilogram per day vs. ≥ 3.0 kilocalories per kilogram per day, OR=1.31, 95% CI=1.09-1.59; <1.5 kilocalories per kilogram per day vs. ≥ 3.0 kilocalories per kilogram per day, OR=1.99, 95% CI=1.67-2.36. | OR and 95% CI for poorer SRH.  M: Recreational screen time (> 2 h/d vs.≤ 2h/d), OR=1.28, 95% CI=1.08-1.52. F: Recreational screen time (> 2 h/d vs.≤ 2h/d), OR=1.32, 95% CI=1.13-1.54. |
| Badura et al., 2015 | (1) Individual sports and (2) team sports: organized leisure-time activities participation was measured by 6 items dealing with individual types of activities (team sports, individual sports, art school, youth organizations, recreation/leisure centres, church meeting/singing – including country-specific examples). The dichotomous question “In your free time, do you do any of these organized activities?” with response categories yes/no was followed by the explanatory text: “We mean activities you do in sports or other club or organization”. | NA | “Would you say your health is ...?” with 4 response categories Excellent / Good / Fair / Poor. |  | OR and 95% CI for bettert SRH.  Individual sports (yes vs. no), OR=2.67, 95% CI=2.07-3.44; team sports (yes vs. no), OR=2.3, 95% CI=1.81-2.92 | NA |
| Moor et al., 2014 | MVPA: MVPA was measured, and coded as: 0 = At least 60 min on at least 5 days, 1 = Fewer days. | (1) TV, (2) playing computer games, and (3) using computer: SB included: watching TV, playing computer games, using computer (emails, homework, etc): coded as 0=<2 hours ,1= ≥2 hours daily. | “Would you say your  health is…” with answer categories of (1) excellent; (2) good; (3) fair; (4) poor. |  | OR and 95% CI for poorer SRH.  MVPA (≥ 1 h/d, ＜5 d/wk vs. ≥ 5 d/wk, OR=1.74, 95% CI =1.67-1.81. | OR and 95% CI for poorer SRH.  TV (> 2 h/d vs. <2 h/d), OR=1.31, 95% CI =1.27-1.36. Computer games (> 2 h/dvs. <2 h/d), OR=1.30, 95% CI =1.24-1.35 Computer (including homework) (> 2 h/dvs. <2 h/d), OR=1.03, 95% CI = 1.02-1.03. |
| Herman et al., 2014 | PA was measured with the ActiGraph 7164 accelerometer (ActiGraph, Pensacola, FL). Children were instructed to wear the accelerometer on a belt over their right hip during all waking hours for 7 days, with removal for bathing or swimming.  (1) Light PA, (2) VPA and (3) MVPA: light, moderate, and VPA were defined by the ActiGraph cut-offs proposed by Evenson et al for use in children, whereby 100 cpm < LPA < 2296 cpm, 2296 cpm ≤ MPA < 4012 cpm, and VPA ≥ 4012 cpm. Min spent in MVPA was calculated as the sum of MPA and VPA min. MVPA was dichotomized based on the Canadian PA guidelines for children ages 5–11, recommending a minimum of 60 min of MVPA daily,an approximation was used whereby children achieving a mean of 60 min or more, MVPA per day over all valid accelerometer days were deemed to have met the guidelines. | (1) SB: SB was measured with the ActiGraph 7164 accelerometer (ActiGraph, Pensacola, FL). SB was defined as time spent (in min) ≤ 100 cpm;  (2) TV, (3) computer/video, (4) homework and (5) reading were measured using self- report questionnaire: “Usually, how many hours per day do you spend watching television (including movies); using the computer (including internet) for fun or playing video games (including Nintendo, Gameboy, etc); doing homework (including homework on the computer); reading comics, novels, or other books for fun—on weekdays? On weekend days?” All SB variables were also dichotomized: homework and reading at ≥ 1 h/d vs. < 1 h/d and TV, computer/video at > 2 h/d vs. ≤ 2 h/d. | “In general, is your health excellent, mostly good, or not very good?” |  | OR and 95% CI for poorer SRH or means and *p* value for SRH.  M:  MVPA (< 1 h/d vs. ≥ 1 h/d), OR=2.23, 95% CI =1.31-3.79. VPA (min/d), means for excellent SRH: 20.5, mostly good/not very good SRH: 14.0, *p* <0 .05. Light PA (min/d), means for excellent SRH: 402.8, mostly good/not very good SRH: 403.6, *p* >0 .05. F: MVPA (< 1 h/d vs. ≥ 1 h/d), OR=1.02, 95% CI =0.47-2.23. VPA (min/d), means for excellent SRH: 10.7, mostly good/not very good SRH: 10.0, *p* >0 .05. Light PA (min/d), means for excellent SRH:408.6, mostly good/not very good SRH: 407.7, *p* >0 .05. | OR and 95% CI for poorer SRH.  M:  Video games/computer (> 2 h/d vs. ≤ 2 h/d), OR=1.33, 95% CI =0.78-2.24.  Reading (> 1 h/d vs. ≤ 1 h/d), OR=1.52, 95% CI =0.78-2.97.  TV (> 2 h/d vs. ≤ 2 h/d), OR=0.88, 95% CI =0.53-1.47. Homework (> 1 h/d vs. ≤ 1 h/d), OR=0.96, 95% CI =0.48-1.93. SB (highest tertiles vs. low tertiles), OR=1.26, 95% CI =0.69-2.28. F:  Video games/computer (> 2 h/d vs. ≤ 2 h/d), OR=2.24, 95% CI =1.06-4.72.  Reading (> 1 h/d vs. ≤ 1 h/d), OR=0.38, 95% CI =0.16-0.86.  TV (> 2 h/d vs. ≤ 2 h/d), OR=1.24, 95% CI =0.69-2.23. Homework (> 1 h/d vs. ≤ 1 h/d), OR=1.05, 95% CI =0.56-1.97. SB (highest tertiles vs. low tertiles), OR=0.6, 95% CI =0.32-1.13. |
| Spein et al., 2013 | (1) PA (out of school VPA) for Sami: “Out of school hours: how many times per week do you take part in sport/do physical exercise to the extent that you get out of breath or sweat?” Categories:0,1,2,3,4,5,. . .. (2) PA (VPA) for Inuit: “How often do you exercise hard (running, soccer, something else)” Categories: Every day, At least once a week, Less than weekly and Never; Frequent (1 or more times a week or every day) vs. Seldom (0 times a week, less than weekly or never). | NA | For Sami: “What is your present state of health?” with reply alternatives: poor, not so good, good and very good.  For Inuit: “How would you rate your health?” with five reply alternatives: very poor, poor, fair, good and very good. |  | OR and 95% CI for poorer SRH.  Out-of-school VPA (≥ 1 times/wk vs. 0 times/wk) in Sami, OR=0.21, 95% CI=0.07-0.63. VPA (everyday vs. less than weekly or never) in Inuit, OR=0.5, 95% CI=0.31-0.81. | NA |
| Afridi et al., 2013 | MVPA: physical inactivity was assessed: < 30 min of MVPA for less than 4 days in last 7 days. | NA | “In general, are you satisfied with your health?” recorded on 4 point Likert scale. |  | OR and 95% CI for poorer SRH.  MVPA (< 3 d/wk vs. ≥ 3 d/wk), OR=1.23, 95% CI=0.78-1.95. | NA |
| Tabak et al., 2012 | MVPA: the young people were asked to report the number of days over the past week that they had been physically active for at least 60 min per day. | (1) TV and (2) computer: the participants were asked how many hours per day they watched television (including videos and DVDs) and used a computer (for playing games, emailing, chatting or surfing the internet) in their spare time on weekdays and weekends. The total number of hours they spent during the week in front of a TV or computer was calculated based on these answers. | “Would you say your health is...” with response options of ‘excellent’ (1), ‘very good’ (2), ‘good’ (3), ‘fair’ (4), and ‘poor’ (5). |  | β, SE and *p* value for poorer SRH.  MVPA: Rural, β = -0.14, SE=0.03, *p*<0.001; Urban, β = -0.06, SE=0.03, *p*<0.05) | β, SE and *p* value for poorer SRH.  TV: Rural, β = 0, SE=0.01, *p*>0.05; Urban, β = 0, SE=0.01, *p*>0.05. Computer: Rural, β = -0.01, SE=0.01, *p*>0.05; Urban, β = -0.01, SE=0.01, *p*>0.05. |
| Richter et al., 2012 | MVPA: PA was assessed with a 60 min MVPA screening measure (physically active for at least 60 min on at least 5 days vs. fewer days) | NA | “Would you say your health is ...” with four response options: (1) Excellent; (2) Good; (3) Fair; (4) Poor. |  | OR and 95% CI for poorer SRH.  M: MVPA (≥ 1 h/d, <6 d/wk vs ≥ 6 d/wk), OR=1.23, 95% CI =0.97-1.56. F: MVPA (≥ 1 h/d, <6 d/wk vs ≥ 6 d/wk), OR=1.40, 95% CI =1.09-1.78 | NA |
| Zullig et al., 2011 | (1) VPA, (2) physical education and (3) sports team: PA was measured by the number of days (0, 1 or more) attending vigorous exercise, physical education classes in past 7 days, a sports team (yes/no). PA was categorized into two groups: 0 days/week, ≥1days. | TV: SB was measured by watching TV in an average school day with two categories: less than 1hour, 1 hour or more. | “In general, how would you describe your health?” with five response options: ‘Excellent’, ‘Very good’, ‘Good’, ‘Fair’, and ‘Poor’. |  | OR and 95% CI for poorer SRH.  M: Sports team (no vs. yes), OR=5.40, 95% CI=1.30-22.39. VPA (0 d/wk vs. ≥ 1 d/wk), OR=5.91, 95% CI=0.91-38.31.  Physical education (0 d/wk vs. ≥ 1 d/wk), OR=0.91, 95% CI=0.24-3.46.  F: Sports team (no vs. yes), OR=30.92, 95% CI=3.74-255.43. VPA (0 d/wk vs. ≥ 1 d/wk), OR= 2.89, 95% CI=0.53-15.61. Physical education (0 d/wk vs. ≥ 1 d/wk), OR=2.31, 95% CI=0.64-8.30. | OR and 95% CI for poorer SRH.  M: TV (≥1 h/d vs. < 1h/d), OR=2.42, 95% CI=0.29-20.20. F: TV (≥1 h/d vs. < 1h/d), OR=0.51, 95% CI=0.14-1.85. |
| Foti et al., 2010 | MVPA: PA was assessed by: 1. did not meet recommended levels of PA during the seven days before the survey. | (1) TV and (2) computer: SB included: watched television and used computers ≥3 hours per day. | “How would you describe your health in general?” Response options included “excellent,” “very good,” “good,” “fair,” or “poor.” |  | OR and 95% CI for poorer SRH.  M: MVPA (< 1h/d vs. ≥1 h/d), OR=1.76, 95% CI=1.09-2.84. F:  MVPA (< 1h/d vs. ≥1 h/d), OR=1.35, 95% CI=0.92-1.99. | OR and 95% CI for poorer SRH.  TV (≥3 h/d vs. <3h/d): Non-Hispanic white, OR=1.39, 95% CI=1.11-1.74; Non-Hispanic black, OR=1.10, 95% CI=0.77-1.57; Hispanic, OR=1.36, 95% CI=1.00-1.85. Computers (≥3 h/d vs. <3h/d): Non-Hispanic white, OR=1.70, 95% CI=1.22-2.37; Non-Hispanic black, OR=1.34, 95% CI=0.92-1.94; Hispanic, OR=1.56, 95% CI=1.18-2.07. |
| Joensuu et al., 2024 | (1) Self-reported PA: “Over the past 7 days, on how many days were you physically active for a total of at least 60 min per day?”. The scale ranged from 0 (0 days) to 7 (7 days).  (2) Accelerometer-based PA: the data were collected using a hip-worn accelerometer (ActiGraph GT3X+, wGT3X+, Pensacola, Florida, USA) during a 7-day measurement period. Data were collected at 30 Hz and a 15 second epoch conversion was used for subsequent analysis. Evenson et al criteria were used to MVPA. A valid data required at least 500 min of monitored data per day (between 7:00 and 23:00 hours), including at least 2 weekdays and 1 weekend day. | NA | “What do you think about your health? It is…? Very good, good, fair or poor”. |  | β and *p* value for better SRH.  M: Self-reported PA in 2013 and SRH in 2015, β=0.289, *p*=0.003. Accelerometer-based MVPA showed no longitudinal associations with SRH when adjusted with overall fitness index (articles did not show results).  F: Self-reported PA in 2013 and SRH in 2015, β=0.056, *p*=0.430. Accelerometer-based MVPA showed no longitudinal associations with SRH when adjusted with overall fitness index (articles did not show results). | NA |
| Nigg et al., 2015 | MVPA: children indicated the number of days per week and the number of min per day they engaged in strenuous and moderate PA. Min per day of PA were given in 10-min increments starting with 0 min and ending with 60+min. | SB (recreational screen time): children were asked to report the number of hours per day that they watch TV, play videogames, and use the Internet (not for homework). Responses were given using whole hours from 0 to 10+hours. | “In general, I would say that my health is...” responses were given on a five-point scale where 1 = “poor” and 5 = “excellen.” |  | β and *p* value for better SRH.  MVPA (MVPA in year 1 and SRH in year 5), β=0.01, *p* > 0.05 | β and *p* value for better SRH.  Recreational screen time (Recreational screen time in year 1 and SRH in year 5), β =−0.04, *p* > 0.05 |
| Liu et al., 2015 | Outdoor PA: contents of the self-administered questionnaire on children’s outdoor PAwere examined in phases 2 and 4, frequency of participating in outdoor PA during 1 week, rated on 4-point scale (1: ‘very often’ to 4 : ‘never’). Frequency of PA was recoded as frequent (combining ‘very often’ and ‘often’) or infrequent (combining ‘occasionally’ and ‘never’). | NA | “During the past 4 weeks, how would you rate your health in general?” The response options were ‘excellent’, ‘very good’, ‘good’, ‘fair’, and ‘poor’. |  | OR and 95% CI for better SRH.  M: Reporting participating in outdoor PA (persisting participate vs. no) at both ages 6 and 12 years were associated with higher likelihood of good SRH at aged 12, OR=1.47, 95% CI=1.14-1.89. F: Reporting participating in outdoor PA (persisting participate vs. no) at both ages 6 and 12 years were not associated with higher likelihood of good SRH at aged 12, OR=1.14, 95% CI=0.92-1.42. | NA |
| Spengler et al., 2014 | PA: adolescents were asked about frequency, duration and type of their weekly habitual PA in the settings sports club and leisure time outside of sports clubs. | Media use (recreational screen time): media use was assessed in the KiGGS Survey with a questionnaire asking the adolescents about the daily amount of time they spend on watching TV, using a computer and playing console games. The answers were coded with the following values: ‘never’ = 0,‘approx. 30 min’ = 0.5, ‘one to two hours’ = 1.5, ‘three to four hours’ = 3.5, ‘more than four hours’ = 5. | Participants were asked how they would rate their state of health in general. Answer categories were “very good”, “good”, “fair”, “poor” and “very poor”. |  | Means and *p* value for SRH.  M: PA, means for poor SRH, time 1 (2003-2006): 1.81, time 2 (2009-2012): 1.58, *p*<0.05. F: PA, means for poor SRH, time 1 (2003-2006): 1.71, time 2 (2009-2012): 1.84, *p*>0.05. | Means and p value for SRH.  M:  Recreational screen time, means for poor SRH, time 1 (2003-2006): 1.89, time 2 (2009-2012):1.83, *p*>0.05. F:  Recreational screen time, means for poor SRH, time 1 (2003-2006): 2.19, time 2 (2009-2012): 2.10, *p*>0.05. |
| Jerdén et al., 2011 | (1) Physical exercis (out of school VPA): “How often do you usually exercise in your spare time (i.e. outside school) so you become breathless or sweating?”, using a 7-grade ordinal scale. An answer of “two or three times weekly” or “more often” was regarded as “high.”  (2) Other PA (out of school PA): “How often do you do other things on your spare time (i.e., outside school) that increases your physical fitness, as walking, cycling, roller blades, dancing etc?”, using a 7-grade ordinal scale. The answer of ‘two or three times weekly’ or ‘more often’ was classified as ‘high’, and the remaining answer as ‘low’ for both physical exercise and other PA. | NA | “How do you consider your well-being most of the time?”, using a 5 grade ordinal scale. “very good,” “rather good,” “nor good nor bad,” “rather bad,” or “very bad”. |  | OR and 95% CI for better SRH.  M: Out-of-school VPA (> 3times/wk vs. ≤3times/wk) in grade 7 and SRH in grade 9, OR=1.17, 95% CI =1.02-1.35. Out-of-school PA (> 3times/wk vs. ≤3times/wk) in grade 7 and SRH in grade 9, OR=1.09, 95% CI=0.94-1.25. F: Out-of-school VPA (> 3times/wk vs. ≤3times/wk) in grade 7 and SRH in grade 9, OR=1.20, 95% CI=1.03-1.39. Out-of-school PA (> 3times/wk vs. ≤3times/wk) in grade 7 and SRH in grade 9, OR=1.13, 95% CI=0.97-1.30. | NA |
| Elinder et al., 2011 | PA (VPA): “How many hours per week, in your leisure time, do you move so intensively that you become out of breath or sweat?” Six predetermined options were given, from <1⁄2 hour to >6 hours. Leisure time PA was then divided into three categories as follows: >4 hours per week, 2–4 hours, and < 2 hours per week. | NA | “How healthy do you feel?” This question could be answered by three options, “very healthy,” “quite healthy,” and “not very healthy.” |  | OR and 95% CI for poorer SRH.  M: VPA in grade 9 and SRH in grade 12: <2 h/d vs. > 4h/d, OR=14.69, 95% CI=1.69-127.13; 2–4h/d vs. > 4h/d, OR=16.04, 95% CI=2.09-123.33. F: VPA in grade 9 and SRH in grade 12: <2 h/d vs. > 4h/d, OR=1.56, 95% CI=0.72-3.37; 2–4 h/d vs. > 4 h/d, OR=0.99, 95% CI=0.49-1.99. | NA |

Notes: PA, physical activity; SB, sedentary behavior; SRH, self-rated health; VPA, vigorous physical activity; MVPA, moderate to vigorous physical activity; MET, metabolic equivalent of task; M, male; F, female; NA, not applicable; min, minutes; h/d, hour/day; h/wk, hour/week; d/wk, day/week; times/wk, times/week; min/wk, minutes/week.

| Supplementary Table 4. Summary of associations between physical activity and self-rated health by frequency/duration | | | | | | | |  |
| --- | --- | --- | --- | --- | --- | --- | --- | --- |
| Variable | Frequency/  duration | Literatures | Association | Summary | | | | % |
|  |  |  |  | # of subgroup | + | - | O |  |
| VPA | Everyday | (Spein et al.,2013)^(everyday) in Inuit^; (Lachytova et al.,2017)^(everyday)^; (Liang et al., 2024)M^(> 1 h/d)^; (Liang et al., 2024)F^(> 1 h/d)^ | + | 4 | 4 | 0 | 0 | 100% (4/4) |
|  | ≥ 3 d/wk | (Hwang et al.,2018)^(≥ 3 d/wk)^; (Spein et al.,2013)^(everyday) in Inuit^; (Lachytova et al.,2017)^(everyday)^; (Liang et al., 2024)M^(> 1 h/d)^; (Liang et al., 2024)F^(> 1 h/d)^ | + | 5 | 5 | 0 | 0 | 100% (5/5) |
|  | ≥ 2 d/wk | (Jodkowska et al.,2019)F^(≥ 2 d/wk) out-of-school VPA^; (Pierannunzio et al.,2022)F^(≥ 2 d/wk) out-of-school VPA^ ^in 11 year old^; (Pierannunzio et al.,2022)F^(≥ 2 d/wk) out-of-school VPA^ ^in 13 year old^; (Pierannunzio et al.,2022)F^(≥ 2 d/wk) out-of-school VPA^ ^in 15 year old^; (Pierannunzio et al.,2022)M^(≥ 2 d/wk) out-of-school VPA^ ^in 13 year old^; (Pierannunzio et al.,2022)M^(≥ 2 d/wk) out-of-school VPA^ ^in 15 year old^; (Hwang et al.,2018)^(≥ 3 d/wk)^; (Spein et al.,2013)^(everyday) in Inuit^; (Lachytova et al.,2017)^(everyday)^; (Liang et al., 2024)M^(> 1 h/d)^; (Liang et al., 2024)F^(> 1 h/d)^ | + | 12 | 11 | 0 | 1 | 92% (11/12) |
|  |  | (Pierannunzio et al.,2022)M^(≥ 2 d/wk) out-of-school VPA^ ^in 11 year old^ | O |  |  |  |  |  |
|  | ≥1 d/wk | Jodkowska et al.,2019)F^(≥ 2 d/wk) out-of-school VPA^; (Pierannunzio et al.,2022)F^(≥ 2 d/wk) out-of-school VPA^ ^in 11 year old^; (Pierannunzio et al.,2022)F^(≥ 2 d/wk) out-of-school VPA^ ^in 13 year old^; (Pierannunzio et al.,2022)F^(≥ 2 d/wk) out-of-school VPA^ ^in 15 year old^; (Pierannunzio et al.,2022)M^(≥ 2 d/wk) out-of-school VPA^ ^in 13 year old^; (Pierannunzio et al.,2022)M^(≥ 2 d/wk) out-of-school VPA^ ^in 15 year old^; (Hwang et al.,2018)^(≥ 3 d/wk)^; (Spein et al.,2013)^(everyday) in Inuit^; (Lachytova et al.,2017)^(everyday)^; (Liang et al., 2024)M^(> 1 h/d)^; (Liang et al., 2024)F^(> 1 h/d)^ | + | 14 | 11 | 0 | 3 | 79% (11/14) |
|  |  | (Zullig et al.,2011)M^(≥1d/wk)^; (Zullig et al.,2011)F^(≥1d/wk)^; (Pierannunzio et al.,2022)M^(≥ 2 d/wk) out-of-school VPA^ ^in 11 year old^ | O |  |  |  |  |  |
|  | 7 times/wk | (Wang et al.,2022)^(7 times/wk)^ | + | 1 | 1 | 0 | 0 | 100% (1/1) |
|  | ≥ 4 times/wk | (Jerdén et al.,2011)M^(> 3times/wk) out-of-school VPA^; (Jerdén et al.,2011)F^(> 3times/wk)^; (Lachytova et al.,2017)^(4–6 times/wk)^; (Wang et al.,2022)^(7 times/wk)^ | + | 4 | 4 | 0 | 0 | 100% (4/4) |
|  | ≥ 2 times/wk | (Jerdén et al.,2011)M^(> 3times/wk) out-of-school VPA^; (Jerdén et al.,2011)F^(> 3times/wk)^; (Lachytova et al.,2017)^(4–6 times/wk)^; (Wang et al.,2022)^(7 times/wk)^ |  | 5 | 4 | 0 | 1 | 80% (4/5) |
|  |  | (Lachytova et al.,2017)^(2–3 times/wk)^ | O |  |  |  |  |  |
|  | ≥ 1 times/wk | (Spein et al.,2013)^(≥ 1 times/wk) out-of-school VPA in Sami^; (Jerdén et al.,2011)M^(> 3times/wk) out-of-school VPA^; (Jerdén et al.,2011)F^(> 3times/wk)^; (Lachytova et al.,2017)^(4–6 times/wk)^; (Wang et al.,2022)^(7 times/wk)^ | + | 7 | 5 | 0 | 2 | 71% (5/7) |
|  |  | (Lachytova et al.,2017)^(1 times/wk)^; (Lachytova et al.,2017)^(2–3 times/wk)^ | O |  |  |  |  |  |
|  | > 4 h/wk | (Elinder et al.,2011)M^(> 4 h/wk)^ | + | 2 | 1 | 0 | 1 | 50% (1/2) |
|  |  | (Elinder et al.,2011)F^(> 4 h/wk)^ | O |  |  |  |  |  |
|  | 2-4 h/wk | (Elinder et al.,2011)M^(> 4 h/wk)^ | + | 2 | 1 | 0 | 1 | 50% (1/2) |
|  |  | (Elinder et al.,2011)F^(> 4 h/wk)^ | O |  |  |  |  |  |
| MVPA | ≥ 1 h/d, everyday | (Herman et al.,2014)M^(≥ 1 h/d)^; (Jodkowska et al.,2019)F^(≥ 1 h/d)^; (Foti et al.,2010)M^(≥ 1 h/d^); (Granger et al.,2017)^(≥ 1 h/d)^; (de Fátima Guimarães et al.,2022)F^(≥ 1 h/d)^; (Kyan et al.,2022)^(≥ 1 h/d) junior high school students^; (Karchynskaya et al.,2022)^(≥ 1 h/d, 7 d/wk)^; (Moran et al., 2024)^(≥ 1 h/d, 7 d/wk)^; (Liang et al., 2024)M^(≥ 1 h/d) weekdays and weekend^*****; (Liang et al., 2024)F^(≥ 1 h/d) weekdays and weekend^*****; (Shi et al., 2023)^(≥ 1 h/d)^ | + | 14 | 11 | 0 | 3 | 79% (11/14) |
|  |  | (Herman et al.,2014)F^(≥ 1 h/d)^; (Foti et al.,2010)F^(≥ 1 h/d)^; (Kyan et al.,2022)^(≥ 1 h/d) elementary school students^ | O |  |  |  |  |  |
|  | ≥ 1 h/d, ≥ 6 d/wk | (Richter et al.,2012)F^(≥ 1 h/d, ≥ 6 d/wk)^; (Herman et al.,2014)M^(≥ 1 h/d)^; (Jodkowska et al.,2019)F^(≥ 1 h/d)^; (Foti et al.,2010)M^(≥ 1 h/d^); (Granger et al.,2017)^(≥ 1 h/d)^; (de Fátima Guimarães et al.,2022)F^(≥ 1 h/d)^; (Kyan et al.,2022)^(≥ 1 h/d) junior high school students^; (Karchynskaya et al.,2022)^(≥ 1 h/d, 7 d/wk)^; (Moran et al., 2024)^(≥ 1 h/d, 7 d/wk)^; (Liang et al., 2024)M^(≥ 1 h/d) weekdays and weekend^*****; (Liang et al., 2024)F^(≥ 1 h/d) weekdays and weekend^*****; (Shi et al., 2023)^(≥ 1 h/d)^ | + | 16 | 12 |  | 4 | 75% (12/16) |
|  |  | (Richter et al.,2012)M^(≥ 1 h/d, ≥ 6 d/wk)^; (Herman et al.,2014)F^(≥ 1 h/d)^; (Foti et al.,2010)F^(≥ 1 h/d)^; (Kyan et al.,2022)^(≥ 1 h/d) elementary school students^ | O |  |  |  |  |  |
|  | ≥ 1 h/d, ≥ 5 d/wk | (Martinez-Lopez et al.,2015)M^(≥ 1 h/d, > 4 d/wk)^; (Martinez-Lopez et al.,2015)F^(≥ 1 h/d, > 4 d/wk)^; (Moor et al.,2014)^(≥ 1 h/d, ≥ 5 d/wk)^; (Karchynskaya et al.,2022)^(≥ 1 h/d, 5-7 d/wk)^; (Moran et al., 2024)^(≥ 1 h/d, 5-6 d/wk)^; (Richter et al.,2012)F^(≥ 1 h/d, ≥ 6 d/wk)^; (Herman et al.,2014)M^(≥ 1 h/d)^; (Jodkowska et al.,2019)F^(≥ 1 h/d)^; (Foti et al.,2010)M^(≥ 1 h/d^); (Granger et al.,2017)^(≥ 1 h/d)^; (de Fátima Guimarães et al.,2022)F^(≥ 1 h/d)^; (Kyan et al.,2022)^(≥ 1 h/d) junior high school students^; (Karchynskaya et al.,2022)^(≥ 1 h/d, 7 d/wk)^; (Moran et al., 2024)^(≥ 1 h/d, 7 d/wk)^; (Liang et al., 2024)M^(≥ 1 h/d) weekdays and weekend^*****; (Liang et al., 2024)F^(≥ 1 h/d) weekdays and weekend^*****; (Shi et al., 2023)^(≥ 1 h/d)^ | + | 21 | 17 | 0 | 4 | 81% (17/21) |
|  |  | (Richter et al.,2012)M^(≥ 1 h/d, ≥ 6 d/wk)^; (Herman et al.,2014)F^(≥ 1 h/d)^; (Foti et al.,2010)F^(≥ 1 h/d)^; (Kyan et al.,2022)^(≥ 1 h/d) elementary school students^ | O |  |  |  |  |  |
|  | ≥ 1 h/d, ≥ 4 d/wk | (Pierannunzio et al.,2022)M^(≥ 1 h/d, ≥ 4 d/wk) 11 years old^; (Pierannunzio et al.,2022)F^(≥ 1 h/d, ≥ 4 d/wk) 13 years old^; (Pierannunzio et al.,2022)M^(≥ 1 h/d, ≥ 4 d/wk) 13 years old^; (Pierannunzio et al.,2022)F^(≥ 1 h/d, ≥ 4 d/wk) 15 years old^; (Pierannunzio et al.,2022)M^(≥ 1 h/d, ≥ 4 d/wk) 15 years old^; (Martinez-Lopez et al.,2015)M^(≥ 1 h/d, > 4 d/wk)^; (Martinez-Lopez et al.,2015)F^(≥ 1 h/d, > 4 d/wk)^; (Moor et al.,2014)^(≥ 1 h/d, ≥ 5 d/wk)^; (Karchynskaya et al.,2022)^(≥ 1 h/d, 5-7 d/wk)^; (Moran et al., 2024)^(≥ 1 h/d, 5-6 d/wk)^; (Richter et al.,2012)F^(≥ 1 h/d, ≥ 6 d/wk)^; (Herman et al.,2014)M^(≥ 1 h/d)^; (Jodkowska et al.,2019)F^(≥ 1 h/d)^; (Foti et al.,2010)M^(≥ 1 h/d^); (Granger et al.,2017)^(≥ 1 h/d)^; (de Fátima Guimarães et al.,2022)F^(≥ 1 h/d)^; (Kyan et al.,2022)^(≥ 1 h/d) junior high school students^; (Karchynskaya et al.,2022)^(≥ 1 h/d, 7 d/wk)^; (Moran et al., 2024)^(≥ 1 h/d, 7 d/wk)^; (Liang et al., 2024)M^(≥ 1 h/d) weekdays and weekend^*****; (Liang et al., 2024)F^(≥ 1 h/d) weekdays and weekend^*****; (Shi et al., 2023)^(≥ 1 h/d)^ | + | 27 | 22 | 0 | 5 | 81% (22/27) |
|  |  | (Pierannunzio et al.,2022)F^(≥ 1 h/d, ≥ 4 d/wk) 11 years old^; (Richter et al.,2012)M^(≥ 1 h/d, ≥ 6 d/wk)^; (Herman et al.,2014)F^(≥ 1 h/d)^; (Foti et al.,2010)F^(≥ 1 h/d)^; (Kyan et al.,2022)^(≥ 1 h/d) elementary school students^ | O |  |  |  |  |  |
|  | ≥ 1 h/d, ≥ 3 d/wk | (Moran et al., 2024)^(≥ 1 h/d, 3-4 d/wk)^; (Pierannunzio et al.,2022)M^(≥ 1 h/d, ≥ 4 d/wk) 11 years old^; (Pierannunzio et al.,2022)F^(≥ 1 h/d, ≥ 4 d/wk) 13 years old^; (Pierannunzio et al.,2022)M^(≥ 1 h/d, ≥ 4 d/wk) 13 years old^; (Pierannunzio et al.,2022)F^(≥ 1 h/d, ≥ 4 d/wk) 15 years old^; (Pierannunzio et al.,2022)M^(≥ 1 h/d, ≥ 4 d/wk) 15 years old^; (Martinez-Lopez et al.,2015)M^(≥ 1 h/d, > 4 d/wk)^; (Martinez-Lopez et al.,2015)F^(≥ 1 h/d, > 4 d/wk)^; (Moor et al.,2014)^(≥ 1 h/d, ≥ 5 d/wk)^; (Karchynskaya et al.,2022)^(≥ 1 h/d, 5-7 d/wk)^; (Moran et al., 2024)^(≥ 1 h/d, 5-6 d/wk)^; (Richter et al.,2012)F^(≥ 1 h/d, ≥ 6 d/wk)^; (Herman et al.,2014)M^(≥ 1 h/d)^; (Jodkowska et al.,2019)F^(≥ 1 h/d)^; (Foti et al.,2010)M^(≥ 1 h/d^); (Granger et al.,2017)^(≥ 1 h/d)^; (de Fátima Guimarães et al.,2022)F^(≥ 1 h/d)^; (Kyan et al.,2022)^(≥ 1 h/d) junior high school students^; (Karchynskaya et al.,2022)^(≥ 1 h/d, 7 d/wk)^; (Moran et al., 2024)^(≥ 1 h/d, 7 d/wk)^; (Liang et al., 2024)M^(≥ 1 h/d) weekdays and weekend^*****; (Liang et al., 2024)F^(≥ 1 h/d) weekdays and weekend^*****; (Shi et al., 2023)^(≥ 1 h/d)^ | + | 29 | 23 | 0 | 6 | 79% (23/29) |
|  |  | (Afridi et al.,2013)^(≥ 3 d/wk)^; (Pierannunzio et al.,2022)F^(≥ 1 h/d, ≥ 4 d/wk) 11 years old^; (Richter et al.,2012)M^(≥ 1 h/d, ≥ 6 d/wk)^; (Herman et al.,2014)F^(≥ 1 h/d)^; (Foti et al.,2010)F^(≥ 1 h/d)^; (Kyan et al.,2022)^(≥ 1 h/d) elementary school students^; (Moran et al., 2024)^(≥ 1 h/d, 3-4 d/wk)^; (Pierannunzio et al.,2022)M^(≥ 1 h/d, ≥ 4 d/wk) 11 years old^; (Pierannunzio et al.,2022)F^(≥ 1 h/d, ≥ 4 d/wk) 13 years old^; (Pierannunzio et al.,2022)M^(≥ 1 h/d, ≥ 4 d/wk) 13 years old^; (Pierannunzio et al.,2022)F^(≥ 1 h/d, ≥ 4 d/wk) 15 years old^; (Pierannunzio et al.,2022)M^(≥ 1 h/d, ≥ 4 d/wk) 15 years old^; (Martinez-Lopez et al.,2015)M^(≥ 1 h/d, > 4 d/wk)^; (Martinez-Lopez et al.,2015)F^(≥ 1 h/d, > 4 d/wk)^; (Moor et al.,2014)^(≥ 1 h/d, ≥ 5 d/wk)^; (Karchynskaya et al.,2022)^(≥ 1 h/d, 5-7 d/wk)^; (Moran et al., 2024)^(≥ 1 h/d, 5-6 d/wk)^; (Richter et al.,2012)F^(≥ 1 h/d, ≥ 6 d/wk)^; (Herman et al.,2014)M^(≥ 1 h/d)^; (Jodkowska et al.,2019)F^(≥ 1 h/d)^; (Foti et al.,2010)M^(≥ 1 h/d^); (Granger et al.,2017)^(≥ 1 h/d)^; (de Fátima Guimarães et al.,2022)F^(≥ 1 h/d)^; (Kyan et al.,2022)^(≥ 1 h/d) junior high school students^; (Karchynskaya et al.,2022)^(≥ 1 h/d, 7 d/wk)^; (Moran et al., 2024)^(≥ 1 h/d, 7 d/wk)^; (Liang et al., 2024)M^(≥ 1 h/d) weekdays and weekend^*****; (Liang et al., 2024)F^(≥ 1 h/d) weekdays and weekend^*****; (Shi et al., 2023)^(≥ 1 h/d)^ | O |  |  |  |  |  |
|  | ≥ 1 h/d, ≥ 1 d/wk | (Moran et al., 2024)^(≥ 1 h/d, 1-2 d/wk)^; | + | 30 | 24 | 0 | 6 | 80% (24/30) |
|  |  | (Afridi et al.,2013)^(≥ 3 d/wk)^; (Pierannunzio et al.,2022)F^(≥ 1 h/d, ≥ 4 d/wk) 11 years old^; (Richter et al.,2012)M^(≥ 1 h/d, ≥ 6 d/wk)^; (Herman et al.,2014)F^(≥ 1 h/d)^; (Foti et al.,2010)F^(≥ 1 h/d)^; (Kyan et al.,2022)^(≥ 1 h/d) elementary school students^ | O |  |  |  |  |  |
| Out-of-school PA | Everyday | (Yang et al., 2023)^(> 1 h/d) Housework^ | + | 2 | 1 | 0 | 1 | 50% (1/2) |
|  |  | (Yang et al., 2023)^(0.5–1 h/d) Housework^ | O |  |  |  |  |  |
|  | ≥ 2 d/wk | (Jodkowska et al.,2019)F^(≥ 2 d/wk) out-of-school VPA^; (Pierannunzio et al.,2022)F^(≥ 2 d/wk) out-of-school VPA^ ^in 11 year old^; (Pierannunzio et al.,2022)F^(≥ 2 d/wk) out-of-school VPA^ ^in 13 year old^; (Pierannunzio et al.,2022)F^(≥ 2 d/wk) out-of-school VPA^ ^in 15 year old^; (Pierannunzio et al.,2022)M^(≥ 2 d/wk) out-of-school VPA^ ^in 13 year old^; (Pierannunzio et al.,2022)M^(≥ 2 d/wk) out-of-school VPA^ ^in 15 year^; (Yang et al., 2023)^(> 1 h/d) Housework^ | + | 9 | 7 | 0 | 2 | 78% (7/9) |
|  |  | (Pierannunzio et al.,2022)M^(≥ 2 d/wk) out-of-school VPA^ ^in 11 year old^; (Yang et al., 2023)^(0.5–1 h/d) Housework^ | O |  |  |  |  |  |
|  | > 3times/wk | (Jerdén et al.,2011)M^(> 3times/wk) out-of-school VPA^; (Jerdén et al.,2011)F^(> 3times/wk) out-of-school VPA^ | + | 4 | 2 | 0 | 2 | 50% (2/4) |
|  |  | (Jerdén et al.,2011)F^(> 3times/wk)^; (Jerdén et al.,2011)M^(> 3times/wk)^ | O |  |  |  |  |  |
|  | > 1times/wk | (Spein et al.,2013)^(≥ 1 times/wk) out-of-school VPA in Sami^; (Jerdén et al.,2011)M^(> 3times/wk) out-of-school VPA^; (Jerdén et al.,2011)F^(> 3times/wk) out-of-school VPA^ | + | 6 | 3 | 0 | 3 | 50% (3/6) |
|  |  | (Curtin et al.,2018)^(≥ 1 times/wk) sport outside of school^; (Jerdén et al.,2011)F^(> 3times/wk)^; (Jerdén et al.,2011)M^(> 3times/wk)^ | O |  |  |  |  |  |
| Sport | ≥ 1times/wk | (Curtin et al.,2018)^(≥ 1 times/wk)^ ^after school/Saturday school-based organized sport^; (Curtin et al.,2018)^(≥ 1 times/wk) school-based organized sports^; (Curtin et al.,2018)^(≥ 1 times/wk)^ ^sport outside of school^ | O | 3 | 0 | 0 | 3 | 0% (0/3) |

Note: * findings (Liang et al., 2024)^(≥ 1 h/d) weekdays^ and (Liang et al., 2024) ^(≥ 1 h/d) weekend^, both show a positive association, we combined them into a single finding (Liang et al., 2024)^(≥ 1 h/d) weekdays and weekend^ under the category of MVPA (≥ 1 h/d, everyday).

| Supplementary Table 5. Summary of associations of physical activity and sedentary behavior with self-rated health in longitudinal studies | | | | | | | | | | | | | | |
| --- | --- | --- | --- | --- | --- | --- | --- | --- | --- | --- | --- | --- | --- | --- |
| PA/SB | Literatures | Association | Summary | | | | | | | | | | | |
|  |  |  | All | | | | Boys | | | | Girls | | | |
|  |  |  | # of subgroup | + | - | O | # of subgroup | + | - | O | # of subgroup | + | - | O |
| **PA** |  |  |  |  |  |  |  |  |  |  |  |  |  |  |
| MVPA | (Joensuu et al., 2024)M; (Joensuu et al., 2024)F; (Nigg et al., 2015) | O | 3 | 0 | 0 | 3 | 1 | 0 | 0 | 1 | 1 | 0 | 0 | 1 |
| VPA | (Jerdén et al.,2011)M^(> 3times/wk) out-of-school VPA^; (Jerdén et al.,2011)F^(> 3times/wk) out-of-school VPA^; (Elinder et al.,2011)M^(> 4 h/wk)^ | + | 4 | 3 | 0 | 1 | 2 | 2 | 0 | 0 | 2 | 1 | 0 | 1 |
|  | (Elinder et al.,2011)F^(> 4 h/wk)^ | O |  |  |  |  |  |  |  |  |  |  |  |  |
| Outdoor PA | (Liu et al., 2015)M | + | 2 | 1 | 0 | 1 | 1 | 1 | 0 | 0 | 1 | 0 | 0 | 1 |
|  | (Liu et al., 2015)F | O |  |  |  |  |  |  |  |  |  |  |  |  |
| Out-of-school PA | (Jerdén et al.,2011)M^(> 3times/wk) out-of-school VPA^; (Jerdén et al.,2011)F^(> 3times/wk) out-of-school VPA^ | + | 4 | 2 | 0 | 2 | 2 | 1 | 0 | 1 | 2 | 1 | 0 | 1 |
|  | (Jerdén et al.,2011)F^(> 3times/wk)^; (Jerdén et al.,2011)M^(> 3times/wk)^ | O |  |  |  |  |  |  |  |  |  |  |  |  |
| Unspecified PA | (Joensuu et al., 2024)M; (Spengler et al.,2014)M | + | 4 | 2 | 0 | 2 | 2 | 2 | 0 | 0 | 2 | 0 | 0 | 2 |
|  | (Joensuu et al., 2024)F; (Spengler et al.,2014)F | O |  |  |  |  |  |  |  |  |  |  |  |  |
| Total PA summary |  |  | 15 | 6 | 0 | 9 | 7 | 5 | 0 | 2 | 7 | 1 | 0 | 6 |
| **SB** |  |  |  |  |  |  |  |  |  |  |  |  |  |  |
| Recreational screen time | (Nigg et al., 2015); (Spengler et al.,2014)M; (Spengler et al.,2014)F | O | 3 | 0 | 0 | 3 | 1 | 0 | 0 | 1 | 1 | 0 | 0 | 1 |

Note: (Jerdén et al.,2011)M^(> 3times/wk) out-of-school VPA^; (Jerdén et al.,2011)F^(> 3times/wk) out-of-school VPA^, these instances were only counted once in the final calculation of total PA to avoid double-counting, as they overlap in categories VPA and out-of-school PA.


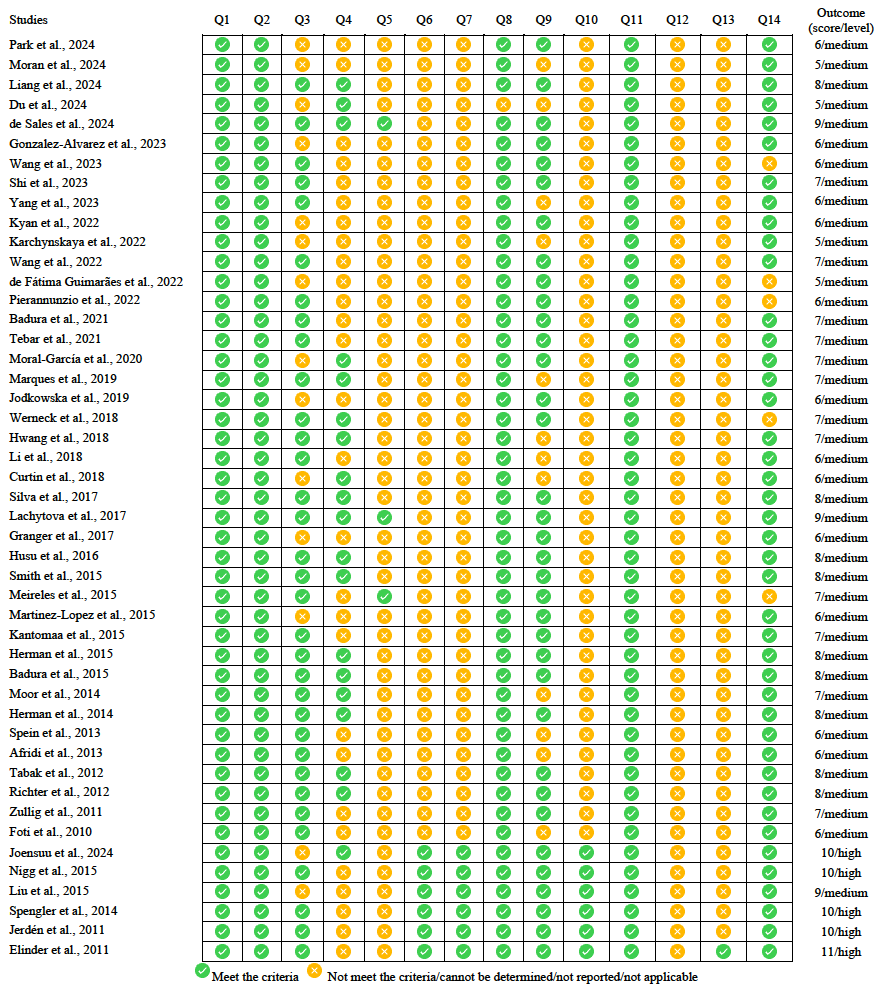


Supplementary Fig. 1 Evaluation of studies’ quality

Note: Q, question.

Q1. Was the research question or objective in this paper clearly stated?

Q2. Was the study population clearly specified and defined?

Q3. Was the participation rate of eligible persons at least 50%?

Q4. Were all the subjects selected or recruited from the same or similar populations (including the same time period)? Were inclusion and exclusion criteria for being in the study prespecified and applied uniformly to all participants?

Q5. Was a sample size justification, power description, or variance and effect estimates provided?

Q6. For the analyses in this paper, were the exposure(s) of interest measured prior to the outcome(s) being measured?

Q7. Was the timeframe sufficient so that one could reasonably expect to see an association between exposure and outcome if it existed?

Q8. For exposures that can vary in amount or level, did the study examine different levels of the exposure as related to the outcome (e.g., categories of exposure, or exposure measured as continuous variable)?

Q9. Were the exposure measures (independent variables) clearly defined, valid, reliable, and implemented consistently across all study participants?

Q10. Was the exposure(s) assessed more than once over time?

Q11. Were the outcome measures (dependent variables) clearly defined, valid, reliable, and implemented consistently across all study participants?

Q12. Were the outcome assessors blinded to the exposure status of participants?

Q13. Was loss to follow-up after baseline 20% or less?

Q14. Were key potential confounding variables measured and adjusted statistically for their impact on the relationship between exposure(s) and outcome(s)?
